# Efficient Matrix-Based Channel Hopping Schemes for Blind Rendezvous in Distributed Cognitive Radio Networks

**DOI:** 10.3390/s18124360

**Published:** 2018-12-10

**Authors:** AbdulMajid Al-Mqdashi, Aduwati Sali, Nor kamariah Noordin, Shaiful J. Hashim, Rosdiadee Nordin

**Affiliations:** 1 Wireless and Photonic Networks Research Centre of Excellence (WiPNEt), Department of Computer and Communication Systems Engineering, University Putra Malaysia, Serdang 43400, Selangor, Malaysia; aduwati@upm.edu.my (A.S.); nknordin@upm.my (N.k.N.); sjh@upm.edu.my (S.J.H.); 2 Faculty of Computer Science and Information Systems, Thamar University, Dhamar 87246, Yemen; 3 Department of Electrical, Electronic and Systems Engineering, Universiti Kebangsaan Malaysia, Bangi 43600, Selangor, Malaysia; adee@ukm.edu.my

**Keywords:** distributed cognitive radio networks, blind rendezvous, channel hopping

## Abstract

Channel rendezvous is an initial and important process for establishing communications between secondary users (SUs) in distributed cognitive radio networks. Due to the drawbacks of the common control channel (CCC) based rendezvous approach, channel hopping (CH) has attracted a lot of research interests for achieving blind rendezvous. To ensure rendezvous within a finite time, most of the existing CH-based rendezvous schemes generate their CH sequences based on the whole global channel set in the network. However, due to the spatial and temporal variations in channel availabilities as well as the limitation of SUs sensing capabilities, the local available channel set (ACS) for each SU is usually a small subset of the global set. Therefore, following these global-based generated CH sequences can result in extensively long time-to-rendezvous (TTR) especially when the number of unavailable channels is large. In this paper, we propose two matrix-based CH rendezvous schemes in which the CH sequences are generated based on the ACSs only. We prove the guaranteed and full diversity rendezvous of the proposed schemes by deriving the theoretical upper bounds of their maximum TTRs. Furthermore, extensive simulation comparisons with other existing works are conducted which illustrate the superior performance of our schemes in terms of the TTR metrics.

## 1. Introduction

Owing to the rapid and exponential growth of wireless devices, spectrum scarcity has become a serious problem in affording the tremendous demand for wireless services. However, several statistical studies and worldwide measurements indicate that most of the licensed spectrum bands are heavily under-utilized (e.g., TV bands) [1]. Therefore, cognitive radio (CR) has emerged as a promising technology for solving the spectrum scarcity and under-utilization problems. In cognitive radio networks (CRNs), the unlicensed users, a.k.a. secondary users (SUs) which are equipped with CRs can sense and opportunistically utilize the idle licensed spectrum bands without causing interference to the bands licensed users, a.k.a. primary users (PUs) [2].

In distributed CRNs such as the CR-based self-organizing ad-hoc and sensor networks, SUs interact directly with each other to establish their communication links without requiring a central coordination entity as in the centralized counterparts [3]. According to its own local observation, each SU is associated with an available channel set (ACS) which contains those channels that are sensed idle from any PU activities during spectrum sensing. To start data transmissions between a pair of SUs, they need to meet each other on a commonly available channel and exchange control messages to setup their communication links. This process is called channel rendezvous, which is a fundamental and vital process for initiating the SUs communications and for coordinating the critical networking functionalities. However, implementing rendezvous on available channels is non-trivial and challenging. The difficulty mainly comes from the fact that before rendezvous, SUs are oblivious of each other’s information and even they might be unaware of each other existence. Accordingly, SUs have no consensus about which common channel they have to switch into simultaneously for achieving rendezvous.

A simple approach that is widely adopted in the literature for rendezvous is the common control channel (CCC), e.g., [4,5,6,7,8,9]. In this approach, one channel which assumed to be globally available for all SUs is dedicated for exchanging control messages. However, the CCC has several drawbacks that can constitute the performance bottleneck such as its susceptibility to long-time blocking when re-occupied by PUs, early saturation by SUs specially under dense environments, or jamming by attackers [10,11,12]. Furthermore, the existence of such a global CCC is practically infeasible in distributed CRNs due to the spectrum heterogeneity among SUs which is caused by the spatial and temporal variations of channel availabilities. Even though multiple CCCs have been used to slightly mitigate these problems [13,14], they unfortunately need additional signaling overhead and long delay for establishing and maintaining them. Moreover, they reduce the number of data channels which degrade the performance.

To overcome the aforementioned drawbacks, Channel hopping (CH) has emerged as an alternative blind rendezvous approach which requires neither CCC nor prior knowledge of the other SUs’ ACSs. In the CH approach, each SU generates its CH sequence independently and keeps hopping on the channels according to the generated CH sequence for achieving rendezvous with its potential neighbors. The rendezvous occurs between a pair of neighboring SUs when they hop simultaneously during the same time slot on a channel that is a commonly available for them. However, several challenges need to be considered while designing the CH scheme which are summarized as follows:

(i) Asynchronous clocks: Some existing CH schemes (e.g., [15,16]) require the presence of time-synchronization between SUs where SUs are assumed to start their CH sequences simultaneously in order to ensure rendezvous. However, in distributed CRNs, it is difficult and unpractical to employ synchronization among spatially dispersed SUs. Moreover, SUs may start their CH at different instants of time. Therefore, the CH-based rendezvous scheme must support the asynchronous scenario.
(ii) Anonymous information: Some existing CH schemes (e.g., [17,18,19]) rely on the distinct IDs of SUs for distinguishing their CH sequences in order to guarantee rendezvous. However, in a distributed CRN, SUs are usually anonymous and they do not possess public IDs. Moreover, SUs can be easily attacked once their IDs are exposed. Therefore, anonymous CH schemes without IDs are favorable.
(iii) Asymmetric ACSs: In the literature, two models are often considered to describe the channel availability for neighboring SUs, the symmetric and asymmetric models. In the symmetric model, SUs have identical ACSs. Meanwhile, SUs have diverse ACSs in the asymmetric mode, but there must be at least one commonly available channel in order to ensure rendezvous. The CH-based scheme is required to work under both models due to their importance in practice [20]. However, establishing rendezvous under the asymmetric model is more difficult due to the fewer common channels.
(iv) Heterogeneous sensing capabilities: Most of the existing CH schemes are homogeneous where they assume that all SUs can sense and access the whole global channel set (GCS). However, due to the inherent limitation and heterogeneity of CR-sensing capabilities, SUs can only sense and detect fractions of the GCS which are usually heterogeneous among SUs [21,22]. Accordingly, CH schemes which design their CH sequence for heterogeneous CRs are more practical.

To evaluate the performance of the CH-based rendezvous scheme, the Maximum and Expected Time-To-Rendezvous (MTTR and ETTR) as well as the Rendezvous Diversity (RD) are the most important performance metrics in the literature. The TTR is defined as the required time for a pair of SUs to achieve channel rendezvous on at least one commonly available channel. However, due to the randomness in the starting time of CH sequences by SUs, the TTR is usually in-equable and hence (MTTR and ETTR) are considered to evaluate the TTR performance [23]. The MTTR indicates the worst case TTR which is important to prove the deterministic rendezvous of the CH scheme. On the other side, the RD is defined as the number of distinct channels at which the pair of SUs can rendezvous.

Obliviously, minimizing MTTR/ETTR will reduce the delay for exchanging the control messages between SUs. Meanwhile, maximizing the RD will improve the scheme robustness against the rendezvous failure on some common channels due to the PU re-appearance. However, designing CH schemes that support all the fully distributed rendezvous criteria mentioned before (i.e., asynchronous, anonymous, asymmetric, and heterogeneous) while improving the above metrics is very challenging.

In spite of the existing literature, the CH-based rendezvous schemes can be categorized according to the SUs’ behavior into two different strategies: asymmetric-role and symmetric-role. In the asymmetric-role strategy, SUs are assumed to have pre-assigned roles as either a sender or a receiver before the start of the rendezvous process. Different roles of SUs follow different procedures to generate the CH sequences. Meanwhile, the symmetric-role strategy have no pre-assigned roles and SUs generate their CH sequences using the same procedure. While the former strategy is optimal in minimizing the TTR, its role-based design limits its applications, for example, the SU can not work as a forwarder during one CH period (i.e., receive packets from one SU and then forward it to another).

On the other hand, each of the above categories can be classified according to the channel information utilized to generate the CH sequence into two classes: Global-Channel-based (GC) and Local-Channel-based (LC) schemes [21,22]. The GC schemes utilize the whole GCS to generate their CH sequences where SUs keep hopping on the available and unavailable channels for attempting rendezvous. This prolongs the TTR of these schemes specially when the number of available channels accounts for a small fraction of the GCS. Even though some of the GC schemes try to enhance their performance by randomly replacing the unavailable channels in their CH sequence with available ones. This replacement strategy is not effective and still results in relatively high MTTR [24]. Meanwhile, the LC schemes generate their CH sequences based on the ACSs which make them more practical in distributed CRNs due to the spatial and temporal variations in channel availability as well as the limitation and heterogeneity of SUs sensing capabilities [21,22,24]. However, the majority of existing LC schemes failed to solve the issue efficiently, where they either rely on unfavorable assumptions and unpractical restrictions to guide rendezvous or still produce long TTR due to the inefficient mathematical tools utilized in their designs.

Therefore, in this paper, efficient LC-based and fully distributed CH schemes are proposed for establishing rendezvous in distributed CRNs. Our main contributions are summarized as follows:

- We propose two matrix-based CH schemes, one asymmetric-role named as Quick and Slow CH QS-CH), and one symmetric-role called Interleaved Quick, Slow, and Fixed CH (IQSF-CH). The proposed schemes utilize only the unrestricted local ACSs for generating their CH sequences and can provide deterministic and fast rendezvous with full RD support.
- We prove the guaranteed rendezvous provided by our schemes through deriving the theoretical upper-bound of their MTTRs under the symmetric and asymmetric channel availability models. Also, we conduct extensive simulations to illustrate their superior performance as compared to state-of-art CH rendezvous schemes.

The rest of this paper is organized as follows. Section 2 reviews the related works on rendezvous. The system model and problem formulation are presented in Section 3. The design and theoretical analysis of the proposed CH schemes are presented in Section 4 and Section 5. Using simulations in Section 6, we evaluate the performance of the proposed schemes and compare them with some existing CH-based rendezvous works. Finally, we conclude the paper in Section 7.

## 2. Related Work

In this section, a detailed review for the state-of-art asynchronous CH rendezvous schemes is presented. As stated before, the existing schemes fall into two categories according to the preassigned-role criteria: asymmetric versus symmetric. The schemes in each category can be further classified based on the utilized channels to generate the CH sequence into GC versus LC schemes.

### 2.1. Asymmetric-Role Rendezvous Schemes

**(i) GC schemes.** The Asynchronous CH (ACH) [25] is a typical GC scheme which utilizes array-based quorum systems to generate the CH sequences. The ACH sequences are generated using an L×L array which is assigned with the *L* global channels in a column-wise manner by the sender and in a span-wise manner by the receiver. The Asymmetric Asynchronous CH (ARCH) scheme in [26] and the Full Diversity role-based CH (FDCH-RB) scheme [27] generate their sender and receiver CH sequences based on the concept of opposite movement of SUs around a ring of *L* channels (i.e., clockwise versus anticlockwise). However, different from ARCH, the receiver in FDCH-RB remains at the last channel in the ring for one additional time slot before starting to jump for the next round. Moreover, the FDCH-RB replaces the unavailable channels in its CH sequences with available ones that are selected randomly. The Periodic CH (PCH) scheme [28] constructs each round of its sender CH sequence by arranging the *L* global channels in an ascending order and then in a descending order while repeating the first channel at the last. Meanwhile, the receiver sequence is generated by staying for 2L−1 time slots on each channel of the *L* channels. While PCH replaces the unavailable channels in its sequences according to the round number, its efficiency is not high specially under the asymmetric model. In [29], the Wait-For-Mommy (WFM) CH is proposed in which the receiver plays the role of “mommy” which cycles through the *L* global channels periodically. Meanwhile, the sender plays the role of “child” which stays at the same channel for *L* time slots to be found by the receiver and then repeatedly hops to another unvisited channel. The FARCH [30] is another GC scheme which follows similar procedures as the WFM for generating its CH sequences when *L* is even. However, the FARCH receiver follows a different strategy when *L* is odd which enhances the TTR performance. Although WFM and FARCH can reduce the MTTR when SUs have identical ACSs, they consume unacceptably long TTR under the asymmetric model since they do not adopt any strategy for replacing the unavailable channels. The Asymmetric Asynchronous (AAsync) scheme [31] uses the primitive roots of a prime number calculated based on *L* to generate its CH sequences. The largest primitive root is selected as the default sequence to generate the receiver CH sequence while the primitive root that can obtain the maximum degree of overlapping with default one is selected for generating the sender CH sequence. However, AAsync requires L+1 to be prime.

**(ii) LC schemes.** The CSAC [24] is a representative LC scheme in which the sender CH sequence is generated as a permutation of $p_{s}$ channels that is repeated periodically, where $p_{s}$ is the smallest prime not smaller than the sender number of available channels $n_{s}$. Meanwhile, the first round of the receiver sequence could be any permutation of its $n_{r}$ available channels which is repeatedly left-shifted by 1 during each sub-sequence in the remaining rounds. However, the MTTR of CSAC is very long specially when $n_{r}$ is not divisible by $p_{s}$. In [32], the Dynamic Quorum-based CH (D-QCH) scheme uses quorum system to generate its CH sequences. The receiver D-QCH sequence is generated using an $n_{r}\times L$ array which is assigned in a row-based manner with the receiver $n_{r}$ available channels. Meanwhile, the D-QCH sender sequence is generated as a permutation of $n_{s}$ local available channels which is repeated periodically during the whole CH period. The Sender-Jump Receiver-Wait (SJ-RW) [23,33] is another LC scheme in which sender cycles through its $n_{s}$ available channels periodically according to a randomly generated permutation. On the other hand, the receiver stays at each available channel for L+1 time slots and then repeatedly hops to another unvisited channel for another L+1 slots. Although D-QCH and SJ-RW can reduce the TTR significantly under the symmetric model, their efficiency is not high under the asymmetric model. This is mainly due to the long stay periods by which their receiver stays on each available channel.

Table 1 compares the above mentioned asymmetric-role schemes as well as our proposed QS-CH scheme in terms of the theoretical MTTR and the full RD support. As will be explained later, the MTTR of QS-CH scheme is irrelevant to *L* and only depends on the number of available channels.

### 2.2. Symmetric-Role Rendezvous Schemes

**(i) GC schemes.** The Jump-Stay (JS) [35] is a typical GC scheme in which each period includes three frames of *P* time slots length, where *P* is the smallest prime not smaller than *L*. The first two are jump frames in which the SU switch continuously on channels while the third one is a stay frame where SU stay on a specific channel. The Enhanced Jump-Stay (EJS) scheme [20] is an improved version of JS, in which the number of jump frames is extended to 3 in each period. EJS provides shorter ETTR/MTTR than JS for the case when SUs own asymmetric ACSs. The Disjoint Relaxed Difference Set based scheme (DRDS) [36] exposes *P* sets for generating its CH sequences which can provide full RD but at the cost of high MTTR. The Enhanced Alternate Hop-and-Wait (E-AHW) scheme [18] generates the CH sequence by adopting an alternate hop and wait approach to ensure rendezvous. However, it exposes SUs’ IDs to construct its CH sequences which is not preferred for anonymous SUs with no explicit IDs in the distributed CRNs. Furthermore, the length of exposed IDs are increased with the increase in number of SUs in the network, which degrades the TTR performance. The Fast Rendezvous CH (FRCH) [37] is another GC scheme in which each CH period is generated by arranging the *L* global channels in an ascending order and then in a descending order with one extra parity channel at the last slot. However, FRCH cannot guarantee rendezvous when $L=\frac{(5+2\alpha )r-1}{2}$ (α is a positive integer and *r* is an odd > 3). In the Short-sequence-based (SSB) scheme [38], the CH sequence whose period equals 2L−1 slots is generated based on a folding line concept which hops in a down-up and top-down manners. However, it fails to guarantee rendezvous when $L=\frac{(3+2\alpha )r+1}{2}$ [39].

In [40], two matrix-based rendezvous schemes (T-CH and D-CH) are proposed where the former is non ID-Based and the latter is ID-Based. The T-CH sequence is generated using a matrix which contains $2L+\lceil \frac{L}{2}\rceil$ jump and stay columns. However, the T-CH requires *L* to be a prime and it does not adopts any replacement strategy to replace the unavailable channels which prolong its TTR. On the other hand, the D-CH sequence is generated by concatenating rows in a matrix which contains λ jump and stay columns as well as one running-column (λ is the length of SU’ ID). Even though the D-CH scheme alleviates some drawbacks of the T-CH. It generates CH sequence with the aid of SUs’ IDs similar to E-AHW. The Symmetric-role Quorum-based CH (S-QCH) [32] is another GC scheme in which the CH sequence is generated using an L(2L+1) matrix that contains h-sub and w-sub columns. Each h-sub-column is filled with a permutation of *L* after replacing the unavailable channels while each w-sub-column is filled by an available channel. A major drawback of S-QCH that degrades its MTTR performance is the big size of its constructed matrix specially when *L* is large.

**(ii) LC schemes.** The Heterogeneous Hopping (HH) [41] and Interlocking CH (ICH) [46] are two pioneering LC schemes. Nevertheless, both of them assumed that each SU can observe a range of consecutive channels, which imposes strict limitation to their applications. The Advanced Heterogeneous CH (A-HCH) [47] and Conversion Based Hopping (CBH) [19] schemes relax this assumption where the SUs’ ID are extended into longer cyclically unique IDs by the former or converted into different bit sequences by the latter to distinguish the CH sequences of SUs. Although A-HCH and CBH can maximize the RD and provides shorter ETTR than ICH, they rely on IDs to ensure rendezvous which is unfavorable in distributed CRNs. The local Padded-Dyck-Path-based scheme (L-PDP) [42] adopts dyck paths in a roundabout manner to generate CH. Nevertheless, it requires the periods of sequences to be co-prime which imposes restrictions on its practicality. The Moving Traversing Pointer (MTP) [21,43] is a complete heterogeneous scheme, in which the CH sequence is constructed based on two pointers (a slow-moving and a fast-moving) that move back and forth to attempt rendezvous. However, the efficiency of MTP is not high, specially for the case when the spectrum is fully available. The ZOS [44] is another heterogeneous scheme in which each SU constructs a seed sequence of length 6⌈logL⌉+1 to combine different types of CH sequences. However, it still produces long TTR due to its large seed and inefficient designed sequences. The Single-radio Sunflower-Set (SSS) scheme [22] generates its CH sequence by utilizing the combinatorial features of the sunflower lemma. In this scheme, an approximation algorithm is developed to construct $p_{i}$ disjoint sunflower sets where $p_{i}$ is the smallest prime $⩾n_{i}$. These sunflower sets are then used for mapping the $n_{i}$ local available channels into the time slots of the CH sequence. However, SSS cannot guarantee rendezvous when it is expected in the dynamic phase of the CH sequences while the pair of SUs hop in the same direction with the same hopping steps [45]. The failure of SSS when ($p_{i}=p_{j}$) is verified when we simulate it even when SUs have identical ACSs; which imposes strict limitations to its applications.

The performance comparisons of the state-of-the-art symmetric-role CH rendezvous schemes as well as our proposed IQSF-CH scheme are summarized in Table 2.

## 3. System Model and Problem Definition

### 3.1. System Model

We consider a distributed CRN consisting of *K* SUs that coexist with several PUs in the same geographical area. The licensed spectrum which is owned by PUs is divided into *L* non-overlapping channels, and hence the global channel set in the network is denoted as C={0,1,2,…,L−1}. Each SU is equipped with a half-duplex cognitive radio transceiver that is capable of sensing the spectrum channels and accessing the idle ones opportunistically. It is assumed that each SU can correctly identify its local available channel sets (ACS) after performing any spectrum sensing method [48,49]. A channel is considered available to the SU if its idle from any PU transmissions (i.e., temporally unoccupied by the co-located PUs). The licensed spectrum is assumed to be slow varying where the channel status is slowly time-varying, e.g., the TV spectrum where the PUs (broadcasting stations) utilize their TV channels only at specific times of the day regularly [22]. Hence, the channel availabilities is assumed to remain unchanged during the rendezvous process.

A time-slotted communication is considered in the network where time is divided into discrete slots that have fixed and equal duration. The slot duration *t* is assumed to be in tens of milliseconds which is sufficient for handshaking and link establishment (e.g., t=10 ms according to IEEE 802.22 [50]). However, to cope with slot boundary misalignment that happen when SUs local clocks are asynchronous, the slot time is prolonged to 2t. In this way, an overlap of *t* is ensured despite any boundary misalignment [32].

In this paper, we mainly focus on the pairwise channel rendezvous between any pair of SUs (say SU${}_{i}$ and SU${}_{j}$) in the CRN. Let $n_{i}$ and $n_{j}$ be the numbers of available channels of SU${}_{i}$ and SU${}_{j}$, respectively. Let $A_{i}=\left\{A_{i}^{1},A_{i}^{2},\ldots ,A_{i}^{n_{i}}\right\}\subseteq C$ and $A_{j}=\left\{A_{j}^{1},A_{j}^{2},\ldots ,A_{j}^{n_{j}}\right\}\subseteq C$ be the two local ACSs of SU ${}_{i}$ and SU${}_{i}$, respectively. In heterogeneous CRNs, each SU may have distinct ACS. Let $\hat{A}=A_{i}\cap A_{j}$ denotes the set of commonly available channels between SUs and let $G=|\hat{A}|$. The pair of SUs can rendezvous with each other only if $\hat{A}\neq \Phi$ (i.e., G≥1).

### 3.2. Rendezvous Problem Formulation

The pairwise channel rendezvous problem is defined as follows: For any two neighbor SUs (SU${}_{i}$ and SU${}_{j}$), we need to generate a CH sequence for each SU according to its own local ACS in order to achieve channel rendezvous between these two SUs within a bounded and short time despite any difference of their CH start times (i.e., asynchronous local clocks). Formally, let $S_{i}=\left\{S_{i}^{1},S_{i}^{2},S_{i}^{3},\cdots ,S_{i}^{T_{i}}\right\}$ and $S_{j}=\left\{S_{j}^{1},S_{j}^{2},S_{j}^{3},\cdots ,S_{j}^{T_{j}}\right\}$ denote the CH sequences for SU${}_{i}$ and SU${}_{j}$, respectively. Also let δ denote the clock drift between SU${}_{i}$ and SU${}_{j}$ in the asynchronous scenario. The channel rendezvous problem can be formulated as:

If $\forall \delta ,\forall A_{i},A_{j},\exists c^{*}\in A_{i}\cap A_{j},s.t.\left(S_{i}^{t}=S_{j}^{\left(t+\delta \right)}=c^{*}\right)$ and $c^{*}$ is available at time slot *t*, then the channel rendezvous is achieved between SU${}_{i}$ and SU${}_{j}$.

The main goal of this paper is to design deterministic pairwise CH scheme for tackling this channel rendezvous problem.

## 4. Quick and Slow Channel Hopping (QS-CH) Scheme

In this section, we present the design for the asymmetric-role CH scheme which is called Quick and Slow Channel Hopping (QS-CH) scheme as well as the analysis of its theoretical performance.

### 4.1. Scheme Design

In this scheme, the pair of SUs attempting to rendezvous are assumed to have different pre-assigned roles which are determined before the start of the rendezvous process. The terms sender and receiver are used in order to differentiate the role of each SU. The basic idea of the QS-CH scheme is to let the sender and receiver hop sequentially on their available channels with different speeds and stay for different time slots on each channel. In particular, the sender will hop on its available channel in a quick manner while the receiver hops on its available channels in a slow manner. However, the period by which the sender hops on all of its available channels and the period by which the receiver stays on each channel of its available channels are designed to be primes. This in order to guarantee their rendezvous when they have different number of available channels.

Algorithms 1 and 2 are used by the sender and receiver, respectively to construct their CH sequences. The generating steps of the QS-CH sequences are as follows.

#### 4.1.1. Sender Sequence in QS-CH

When an SU has data to transmit, it serves as a sender and hence generates a quick hopping sequence (Q-CH) as follows. First, randomly select a hopping offset (*h-offset*) $\in [1,n_{s}]$ which is a coprime with $n_{s}$ (i.e., gcd (*h-offset*, $n_{s}$) = 1) and then invokes Algorithm 1 with the available channels set $A_{s}=\left\{A_{s}^{1},A_{s}^{2},\ldots ,A_{s}^{n_{s}}\right\}$ and *h-offset*. In Algorithm 1, the sender find $p_{s}$ as the smallest prime number which is not smaller than $n_{s}$; this means that if $n_{s}$ is a prime, then $n_{s}=p_{s}$. However, if $n_{s}$ is not prime, then $p_{s}$ is the closest prime number that is larger than $n_{s}$. After that, construct an $n_{s}\times p_{s}$ matrix, called the Sending Matrix (SM) where its columns are filled with the channels in $A_{s}$ according to their corresponding indexes that are determined by the hopping offset. Specifically, the $k_{th}$ column of the matrix when $\left(k⩽n_{s}\right)$ is filled with the channel $A_{s}^{index}$ where index is determined by multiplying the *h-offset* with the column index (i.e., (k−1)) mod $n_{s})+1$). This indicates that the first $n_{s}$ columns in SM are filled with all the available channels based on a permutation that is determined by *h-offset*. Meanwhile, if $\left(k>n_{s}\right)$ which happens when $n_{s}\neq p_{s}$, the $k_{th}$ column of SM is filled with the channel that is assigned to the earlier ${\left(k-n_{s}\right)}_{th}$ column. In other words, the last $\left(p_{s}-n_{s}\right)$ columns in SM are filled with the same channels that are assigned to the first $\left(p_{s}-n_{s}\right)$ columns. Finally, generate the Q-CH sequence which has a length equals $n_{s}p_{s}$ slots by concatenating the matrix rows, row by row.

To this end, we get a Q-CH sequence in which the sender stays for only a single slot on each available channel and then hops to the next one. Thus, each frame of $p_{s}$ slots in the Q-CH sequence contains all the $n_{s}$ available channels. The sender will keep hopping on its available channels according to this sequence until it achieves channel rendezvous with its intended receiver.

**Algorithm 1:** Sender CH generation algorithm (Q-CH).**Input:** Available channels $A_{s}=\left\{A_{s}^{1},A_{s}^{2},\ldots ,A_{s}^{n_{s}}\right\}$, *h-offset*. **Output:**
$S_{s}$, the Q-CH sequence for the sender SU${}_{s}$.
  1:Find $p_{s}$ as the smallest prime that is not smaller than $n_{s}$.  2:Define a Sending Matrix (SM) as an empty matrix of $n_{S}$ rows and $p_{s}$ columns.  3:**for**$k=1:p_{s}$**do**  4: **if** (k⩽ns) **then**  5:  index= ((*h-offset*× (k−1)) mod $n_{s})+1$.  6: **else**  7:  index= ((*h-offset*× ($k-n_{s}-1$)) mod $n_{s})+1$.  8: **end if**  9: SM$\left[1:n_{s}\right]\left[k\right]=A_{s}^{index}$ {Fill the $k_{th}$ column with $A_{s}^{index}$}10:**end for**11:Generate $S_{s}$ as the concatenation of the matrix rows $S_{s}=$ SM$\left[1\right]\left[1:p_{s}\right]\;∥\;$SM$\left[2\right]\left[1:p_{s}\right]\;∥\;\ldots \;∥\;$SM$\left[n_{s}\right]\left[1:p_{s}\right]$12:**return**$S_{s}$.


#### 4.1.2. Receiver Sequence in QS-CH

If an SU has nothing to transmit, it serves as a receiver and generates a slow hopping sequence (S-CH) as follows. Firstly, select randomly a number $\in [1,n_{r}]$ which must be a coprime with $n_{r}$ as the hopping offset (*h-offset*). Then invokes Algorithm 2 with the available channels set $A_{r}=\left\{A_{r}^{1},A_{r}^{2},\ldots ,A_{r}^{n_{r}}\right\}$ and *h-offset*. As illustrated in Algorithm 2, the SU find $p_{r}$ as the smallest even number which is not smaller than $n_{r}$. After that, construct an $n_{r}\times p_{r}$ matrix, called Receiving Matrix (RM) in which the $k_{th}$ row is filled with the channel $A_{r}^{index}$ where *index* is determined by *h-offset*. Finally, generate the S-CH sequence which has a length equals $n_{r}p_{r}$ slots by concatenating the matrix rows, row by row. Accordingly, we get a slow channel hopping sequence where the receiver stays for $p_{r}$ slots on each available channel before it hops to the next channel.

**Algorithm 2:** Receiver CH generation algorithm (S-CH).**Input:**$A_{r}=\left\{A_{r}^{1},A_{r}^{2},\ldots ,A_{r}^{n_{r}}\right\}$, *h-offset*. **Output:**$S_{r}$, the S-CH sequence for the receiver SU${}_{r}$.
1:Find $p_{r}$ as the smallest prime number that is not smaller than $n_{r}$.2:Define a Receiving Matrix (RM) as an empty matrix of $n_{r}$ rows and $p_{r}$ columns.3:**for**$k=1:n_{r}$**do**4: index= ((*h-offset*× (k−1)) mod $n_{r})+1$.5: RM$\left[k\right]\left[1:p_{r}\right]=A_{r}^{index}$ {Fill the $k_{th}$ row with $A_{r}^{index}$}6:**end for**7:Generate $S_{r}$ as the concatenation of the matrix rows $S_{r}=$ RM$\left[1\right]\left[1:p_{r}\right]\;∥\;$RM$\left[2\right]\left[1:p_{r}\right]\;∥\;\ldots \;∥\;$RM$\left[n_{r}\right]\left[1:p_{r}\right]$8:**return**$S_{r}$

**Figure 1:**
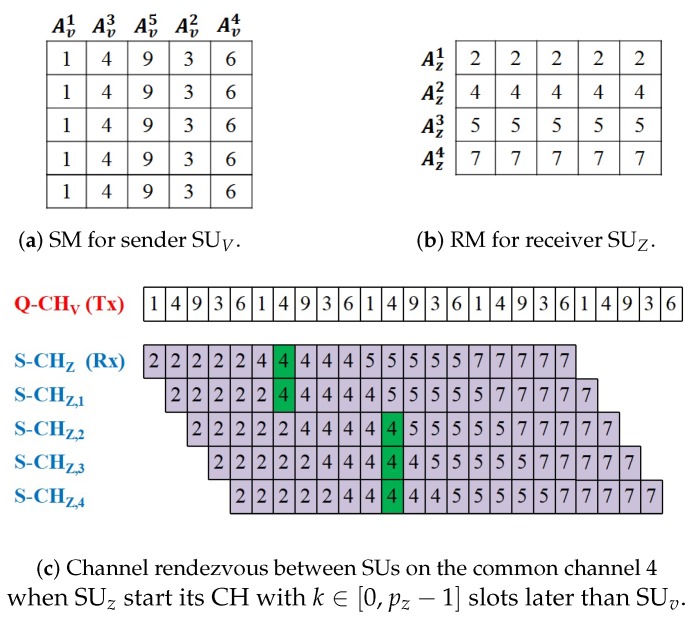
An example of QS-CH sequences constructions and rendezvous for a pair of SUs. (**a**) Sender SU${}_{V}$ has $A_{v}=\left\{1,3,4,6,9\right\}$ and *h-offset* ${}_{v}$= 2. (**b**) Receiver SU${}_{Z}$ has $A_{z}=\left\{2,4,5,7\right\}$ and *h-offset* ${}_{z}$= 1.

Consider the example in Figure 1 which illustrate the generation of the QS-CH sequences for the sender (SU${}_{V}$) and the receiver (SU${}_{Z}$) as well as the rendezvous between them. In Figure 1a, since the number of channels $n_{v}=5$ for the sender SU${}_{V}$ is already prime, hence $p_{v}=n_{v}$ and the sending matrix SM is constructed as an 5×5 matrix. Then, the sender assign its available channels into the columns of the SM according to the selected *h-offset* ${}_{v}$ which is 2. Finally, the sender Q-CH sequence is generated by concatenating the SM rows sequentially which results in a Q-CH with a length of 25 slots. On the other hand, as $n_{z}=4$ is not prime, the receiver SU${}_{Z}$ in Figure 1b obtains $p_{z}=5$ as the smallest prime which is bigger than $n_{z}$. Hence, the RM is constructed as an 5×4 matrix where its rows are filled with the SU${}_{Z}$ available channels according to the selected *h-offset* ${}_{v}$ which is 1. Finally, the CH sequence is generated by concatenating the RM rows one by one which results in a S-CH with a length of 20 slots.

As shown by Figure 1c, during each frame of $p_{v}=5$ consecutive time slots in its Q-CH sequence, the sender hops on all of its 5 available channels. Meanwhile, the receiver SU${}_{z}$ stays for a frame of $p_{z}=5$ consecutive time slots on each channel of its 4 available channels and then switch to the next channel. Thus, for this example, since $p_{v}=p_{z}$, the rendezvous is guaranteed to occur between SUs on their commonly available channel which is 4 despite any drifts of their time clocks.

### 4.2. Scheme Analysis

In this subsection, we study the theoretical performance of the QS-CH under the symmetric and asymmetric channel availability models. Specifically, we prove the guaranteed rendezvous between any arbitrary two SUs performing the QS-CH by deriving the MTTR upper-bound required for achieving a successful channel rendezvous.

**Theorem** **1.** *The MTTR of QS-CH under the symmetric channel availability model is upper-bounded by ($2p_{s}-1$).*

**Figure 2:**
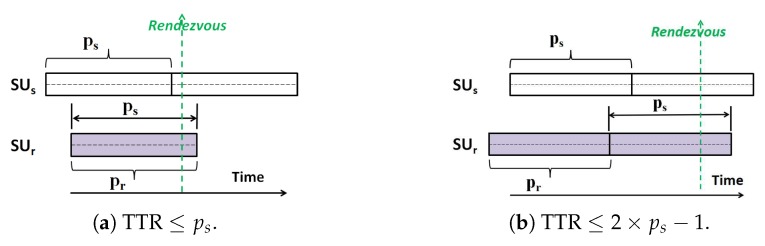
Rendezvous cases for QS-CH under the symmetric channel availability model.

**Proof** **of** **Theorem** **1.** Under the symmetric channel availability model, the sender and receiver SUs always have the same available channels (i.e., $A_{s}\equiv A_{r}$ and $n_{s}\equiv n_{r}=G$). In Figure 2, we present the two possible cases of rendezvous under this model. These cases happen according to the two SUs starting times (i.e., asynchronous scenario).

***Case 1:***Figure 2a. In this sub-case, the sender SU${}_{s}$ starts its CH sequence with $\delta \in [0,n_{s}p_{s}]$ time slots earlier than the start of the receiver SU${}_{r}$. This means that in each S-CH period of SU${}_{r}$, the Q-CH sequence of SU${}_{s}$ is *rotate*( Q-CH${}_{s}$, −δ). As shown by Figure 2a, while the receiver SU${}_{r}$ stays for a frame of $p_{r}$ slots on its first channel, the $p_{s}$ consecutive slots of the sender sequence from the start point of SU${}_{r}$ contains all the sender available channels. According to that and since $A_{s}=A_{r}$, the rendezvous can happen within the first receiver frame. However, since $p_{r}=p_{s}$, we can say that the sender SU can achieve rendezvous with its receiver SU no later than $p_{s}$ slots from the start point of the receiver.

***Case 2:***Figure 2b. In this sub-case, the sender SU${}_{s}$ starts CH with $\delta \in [0,n_{r}p_{r}]$ time slots later than the start of the receiver SU${}_{r}$ which means that in each Q-CH period of SU${}_{s}$, the S-CH sequence of SU${}_{r}$ is *rotate*( S-CH${}_{r}$, δ). In Figure 2b, it is indicated that the sender SU${}_{s}$ does not have enough time slots to hop on all of its available channels before the receiver SU${}_{r}$ transfer to the next channel. So, the rendezvous is only guaranteed during the next frame after the receiver SU${}_{r}$ transfers to the next available channel. Hence, for any value of the misalignment δ, the TTR is guaranteed to occur no later than ($2p_{s}-1$) time slots from the start point of the sender SU${}_{s}$.

To sum up, we approved that under the symmetric channel availability model, the MTTR for rendezvous between any two asymmetric-role SUs (a sender and receiver) which are performing QS-CH is upper bound by $2p_{s}-1$. □

**Theorem** **2.** *Under the asymmetric channel availability model, the MTTR of the QS-CH scheme is given by:*

(1) $$MTTR\leq \left\{\begin{array}{cc} \left(\left(n_{r}-G\right)p_{r}\right)+2p_{s}-1 & if\;p_{s}<p_{r}\\ \left(n_{r}-G+1\right)p_{r} & if\;p_{s}=p_{r}\\ \left(n_{r}p_{r}-G+1\right)p_{s} & if\;p_{s}>p_{r} \end{array}\right.$$

**Proof** **of** **Theorem** **2.** Under the asymmetric channel availability model, the sender and receiver SUs do not have the same ACSs (i.e., $A_{s}\neq A_{r}$). However, the numbers of their available channels ($n_{s}$ and $n_{r}$) may be equal or different. Hence, According to ($n_{s}$ and $n_{r}$) and the corresponding prime numbers ($p_{s}$ and $p_{r}$) obtained by the SUs for generating their CH sequences, we consider the following cases for deriving the theoretical MTTR upper-bound of the QS-CH scheme.

**Figure 3:**
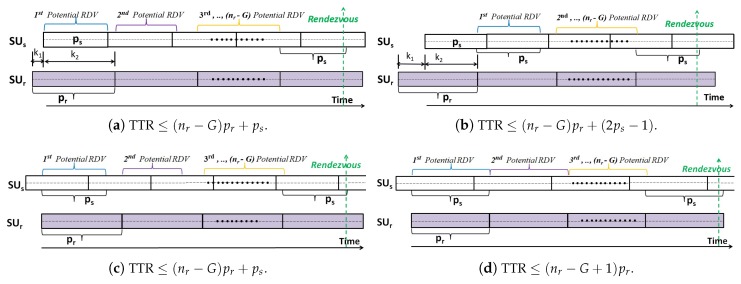
Rendezvous cases for QS-CH under the asymmetric channel availability model when $p_{s}⩽p_{r}$.

***Case 1:***$p_{s}\leq p_{r}$. This case indicates that the frame of the sender QS-CH sequence is smaller or equal to the frame of the receiver SS-CH sequence. In Figure 3, the four possible sub-cases of rendezvous under the asymmetric channel availability model are presented. These four cases happen according to whether $p_{s}<p_{r}$ or $p_{s}=p_{r}$, as well as according to the two SUs starting times (i.e., asynchronous scenario). In particular, the first three figures (Figure 3a–c) occur when $p_{s}<p_{r}$ while the last figure (Figure 3d) occurs when $p_{s}=p_{r}$.

***Case 1.1:*** (Figure 3a). In the figure, $k_{1}$ indicates the clock difference between the SUs start times while $k_{2}$ denotes the remaining time slots of the receiver first frame except $k_{1}$ (i.e., $k_{2}=p_{r}-k_{1}$ ). For this sub-case, $k_{2}\geq p_{s}$ implies that when the sender SU${}_{s}$ starts, it can hop on all of its available channels before $p_{r}$ (i.e., before the receiver SU${}_{r}$ transfers to the next available channel). Hence, the first potential rendezvous can be achieved before $p_{r}$. However, if the channel where the receiver stays is not commonly available between the SUs, the next potential rendezvous can be achieved in the next frame of the receiver SU${}_{r}$ (i.e., from $p_{r}+1$ to $2p_{r}$ time slots). This is because only at the beginning of this frame, the receiver SU${}_{r}$ will transfer to its next available channel and stay on it. The worst case happens when all the first $n_{r}-G$ potential rendezvous times fails because the corresponding receiver stay channels are not commonly available to the sender. After that, starting from the $\left(n_{r}-G\right)p_{r}$ time slot, the receiver SU${}_{r}$ will definitely hop and stays on a channel that is commonly available and hence the rendezvous occurs within $p_{s}$ time slots. Thus, for this case, MTTR $\leq \left(n_{r}-G\right)p_{r}+p_{s}.$

***Case 1.2:*** In this sub-case (Figure 3b), $k_{2}<p_{s}$ implies that the sender SU${}_{s}$ does not have enough time slots to hop on its whole available channels before the receiver SU${}_{r}$ transfer to its next available channel. Accordingly, The first potential rendezvous can be achieved only during the next frame after the receiver SU${}_{r}$ transfers to the next channel. Thereafter, by following a similar analysis of the worst-case as in the previous sub-case, the rendezvous is guaranteed to occur on a common-channel after the failure of at most $\left(n_{r}-G\right)$ potential rendezvous times. So, in this sub-case, MTTR $\leq k_{2}+\left(n_{r}-G\right)p_{r}+p_{s}$ which is less than or equal to $\left(n_{r}-G\right)p_{r}+\left(2p_{s}-1\right)$, since $k_{2}\leq ps-1$.

***Case 1.3:*** In this sub-case (Figure 3c), SU${}_{s}$ starts earlier which indicates that the first potential rendezvous can occur within $p_{s}$ time slots from the starting time of SU${}_{r}$. By following the worst-case analysis similar to the above sub-cases, it can be found that MTTR $\leq \left(n_{r}-G\right)p_{r}+p_{s}.$

***Case 1.4:*** In this sub-case (Figure 3d), $p_{s}=p_{r}$ and the sender SU${}_{s}$ starts earlier. As shown by Figure 3d, while the first frame of the receiver sequence contains its first stay channel, the frame of the sender sequence from the start point of SU${}_{r}$ contains all the sender available channels. Accordingly, the first potential rendezvous can occur within $p_{s}$ time slots from the starting time of SU${}_{r}$. The worst case happens when all the first $\left(n_{r}-G\right)$ potential rendezvous times fails due to the non-availability of receiver stays channels to the sender. However, during the next $p_{r}$ frame, receiver will stay on a commonly available channel and hance a successful rendezvous occurs. So, MTTR $\leq \left(n_{r}-G\right)\times p_{r}+p_{s}$. However, since $p_{s}=p_{r}$, the TTR upper-bound can be represented as MTTR $\leq \left(n_{r}-G\right)p_{r}+p_{r}=\left(n_{r}-G+1\right)p_{r}$.

Above all, it is proved that the MTTR is upper-bound by $\left(n_{r}-G\right)p_{r}+\left(2p_{s}-1\right)$ (when $p_{s}<p_{r}$) as in Case 1.2 since it is the worst case. Meanwhile it is upper-bound by $\left(n_{r}-G+1\right)p_{r}$ (when $p_{s}=p_{r}$).

***Case 2:***$p_{s}>p_{r}$. This case indicates that the frame of $p_{s}$ slots in the sender Q-CH sequence is bigger than the frame of $p_{r}$ slots in the receiver S-CH sequence. Hence, the analysis procedure used in the previous case can not be followed here since the length of each stay frame of the receiver is not large enough to cover one quick frame of the sender.

For this case, since $p_{s}$ and $p_{r}$ are co-primes and $p_{s}>n_{r}$, it can be easily verified that $p_{s}$ and $p_{r}n_{r}$ are also co-primes. For example, suppose that the number of available channels for sender and receiver are $n_{s}=7$ and $n_{r}=4$. This results in $p_{s}=7$ and $p_{r}=5$ which are co-primes. Therefore, $p_{s}=7$ and $p_{r}n_{r}=4\times 5=20$ are also co-primes. According to that and with the help of the Chinese Reminder Theorem [51], it can be proven that within a period of $p_{s}\left(p_{r}n_{r}\right)$ time slots, all the $p_{s}n_{r}$ available channel pairs will be visited for exactly $p_{r}$ times. In other words, let the set which contains all the pairs of available channels for both SUs be $A_{s}\times A_{r}=\left\{\left(A_{s}^{i},A_{r}^{j}\right),i\in \left[1,2,\cdots ,p_{s}\right],j\in \left[1,2,\cdots ,n_{r}\right]\right\}$, where × here indicates the Cartesian product of the two SUs channel sets. If the SUs hops according to their CH sequences for a period of $T=p_{s}\left(p_{r}n_{r}\right)$ slots, then every pair of two channels in $A_{s}\times A_{r}$ will appear for exactly $p_{r}$ times. Thus, the MTTR upper-bound could be represented as $p_{s}\left(p_{r}n_{r}\right)$ for this case. However, this upper-bound is loosely (i.e., not tight) since it is sufficient for the rendezvous guarantee if each pair of two *equal* channels in $A_{s}\times A_{r}$ appears *for one time only*. Thus, it is clear that the MTTR upper-bound can be much less than $p_{s}\left(p_{r}n_{r}\right)$.

Generally, the MTTR depends on the number of common channels *G* as well as on ($p_{s}$ mod $n_{r}p_{r}$) which reflects the deviation amount on the receiver sequence during each repeated frame of the sender sequence. As $p_{s}$ and $n_{r}p_{r}$ are co-primes, the deviation amount ($p_{s}$ mod $n_{r}p_{r})\neq 0$ and is not multiple of $p_{r}$. Hence, deriving the tight theoretical MTTR upper-bound of this case is quit difficult since it requires the consideration for all the possible values of the deviation amount $[0<(p_{s}$ mod $n_{r}p_{r})<n_{r}p_{r}]$. However, we present in (Appendix A), the sub-cases for deriving the MTTR upper-bound under the majority of these values when there is only one commonly channel (i.e., G=1). These sub-cases verify that the MTTR theoretical upper-bound is much less than $p_{s}\left(p_{r}n_{r}\right)$ even when G=1. However, due to the difference in formulas of these sub-cases, the MTTR upper-bound for this case (i.e., when $p_{s}>p_{r}$) is represented for simplicity and unifying purposes as $\left(n_{r}p_{r}-Gp_{r}+1\right)p_{s}$.

Summarizing the discussion above, it is approved that under the asymmetric channel availability model, the MTTR upper bound for rendezvous between any two asymmetric-role SUs (a sender and receiver) which are performing QS-CH is represented by the formulas in Equation (1). □

**Theorem** **3.** *The QS-CH scheme can achieve a full rendezvous diversity (RD) which means that any pair of neighboring SUs which performs QS-CH can rendezvous on all of their commonly available channels.*

**Proof** **of** **Theorem** **3.** Recall the proof of Theorem 2, it is verified that the first successful rendezvous on a commonly available channel can be achieved within a period given by Equation (1). However, for achieving rendezvous over all the *G* commonly available channel, the MTTR upper-bound after eliminating *G* in Equation (1) can be represented as in the following Equation:

(2) $$MTTR\leq \left\{\begin{array}{cc} n_{r}p_{r}+2p_{s}-1 & \mathrm{if}\;p_{s}<p_{r}\\ \left(n_{r}+1\right)p_{r} & \mathrm{if}\;p_{s}=p_{r}\\ \left(n_{r}p_{r}-p_{r}+1\right)p_{s} & \mathrm{if}\;p_{s}>p_{r} \end{array}\right.$$

 □

## 5. Interleaved Quick, Slow, and Fixed Channel Hopping (IQSF-CH) Scheme

In the previous section, the QS-CH scheme was designed using the asymmetric-role approach which requires that each SU has a pre-assigned role (i.e., either a sender or a receiver). In this section, we present the proposed IQSF-CH scheme which is symmetric (i.e., no pre-assigned role assumption).

### 5.1. Scheme Design

In the symmetric-role IQSF-CH scheme, every SU generates its CH sequence with the help of a binary bit sequence called a seed. This seed is generated based on the binary representation of a channel that is selected randomly from the SU ACS. The CH sequence is constructed as an interleaved sequence of several Q-CH and S-CH sequences as well as a fixed stay sequence through a two-step approach. Firstly, based on a certain bit design, each SU transforms the binary representation of the randomly selected available channel into a seed sequence. The resulted seed is composed of several 1’s and 0’s as well as one special bit denoted as “*F*” (short of Fixed). Secondly, each SU replaces any bit of “1” (or “0”) in its seed sequence with a column of Q-based (or S-based) CH sequence. Meanwhile, the SU replaces the last bit of its seed sequence which is denoted as “*F*” with a column filled with its seed channel. To cope with the asynchronous scenario, the seed generation method is designed in such away which ensure that the resulted seed sequences are cyclic rotationally distinct one to the other.

To this end, each SU will construct a matrix of several columns that are filled with quick and slow CH sequences as well as one last column that is filled with the randomly selected seed channel. Accordingly, if the selected seed channels of the two SUs are the same, they generate the same seed which allow them to achieve rendezvous on this channel since they stay on it (i.e., the *F*-type column). Meanwhile, if the seed channels are different which results in generating distinct seeds, the cyclic uniqueness property will guarantee the existence of some time instances where the two SUs are playing different roles. Hence, the channel rendezvous is achieved when one SU is hopping according to its Q-CH sequence while the other SU is hopping based on its S-CH sequence, and visa-versa.

It is worthy mentioning here that IQSF-CH is anonymous rendezvous scheme since it does not rely on the SU’s ID to interleave its sequences such as the works in [17,52]. This makes it more practical for distributed CRNs where SUs do not possess public IDs. Although most of the ID-based works usually adopt different extension methods to transform the SU’s IDs into another cyclic unique IDs for breaking the symmetry, these methods designs are not suitable to generate the seeds in our IQSF-CH due to the existence of F-type bits. Therefore, we propose a new and novel method for generating efficient seed sequences that are suitable to interleave the three types of CH sequences in IQSF-CH.

#### 5.1.1. Generating the Seeds

In this subsection, the method for constructing the seed sequence is presented. For a network of *L* channels, the binary representation for any channel will have a length of m=⌈ log${}_{2}L\rceil$. For example, when the number of channels in the network is L=5, the binary representation for each channel is m=⌈ log${}_{2}5\rceil =3$-bits length. Meanwhile, when L=10, m=⌈ log${}_{2}10\rceil =4$.

In the seed generating method, the *m*-bit binary representation of the seed channel is transformed to a seed sequence by appending it with (m+3) additional bits at the end. The following lemma will illustrate the generation method and its correctness for providing cyclic rotationally distinct seeds.

**Lemma** **1.** *Given any two m-bit binary representations $\alpha =\{\alpha_{1},\cdots ,\alpha_{m}\}$ and $\beta =\{\beta_{1},\cdots ,\beta_{m}\}$. Let **a** and **b** be the two 2m+3-bit expanded seeds generated from α and β as follows:*

$$a\overset{def}{=}\alpha ∥0∥\alpha ∥1∥Fandb\overset{def}{=}\beta ∥0∥\beta ∥1∥F.$$

*The *∥* symbol here indicates the concatenation process. The 0 indicates a bit of zero and 1 indicate a bit of one while F indicate the special type bit. Then **a** and **b** are cyclic rotationally distinct to each other which means that*

a≠rotate(b,k),∀k∈(0,2m+2].

**Figure 4:**
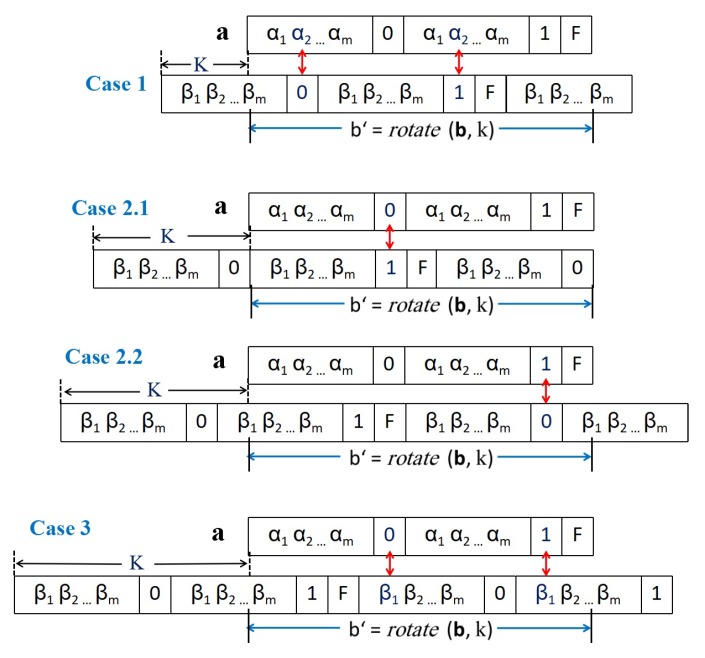
Illustration of the five cases in the proof of Lemma 1.

**Proof** **of** **Lemma** **1.** To prove the above lemma, all the possible cases that may happen when sequence b is rotated with (k∈(0,2m+2]) is considered. In each case, it will be shown that bits in sequence **a** and another bits in sequence b${}^{\prime }=$*rotate*(**b**, k) have different values even though these bits are in the same positions. Considering the three cases in Figure 4 is sufficient to prove that **a** and **b${}^{\prime }$** = *rotate*(**b**, k) are *cyclic rotationally distinct* one to the other.

***Case 1:***k∈(0,m]. As shown by the two red arrows in Figure 4, it holds that $a_{m-k+1}$ is equivalent to $a_{2m-k+2}$ while the same position bits in $b^{\prime }$ have different values (i.e., $b_{m-k+1}^{\prime }=0$ and $b_{2m-k+2}^{\prime }=1$). Hence, if $a_{m-k+1}\equiv a_{2m-k+2}=1$, it implies that $a_{m-k+1}$ is different than $b_{m-k+1}^{\prime }$. However, if $a_{m-k+1}\equiv a_{2m-k+2}=0$, it implies that $a_{2m-k+2}$ is different than $b_{2m-k+2}^{\prime }$. Therefore, in this case, it is proved that there must be at least a single bit that is different in **a** and $b^{\prime }$.

***Case 2.1:***k=m+1. As indicated by the red arrow in Figure 4, it holds that $a_{m+1}=0$ and $b_{m+1}^{\prime }=1$.

***Case 2.2:***k=m+2. As indicated by the red arrow in Figure 4, it holds that $a_{2m+2}=1$ and $b_{2m+2}^{\prime }=0$.

***Case 3:***k∈[2m,2m+2]. As indicated by the two red arrows in Figure 4, it holds that $a_{m+1}=0$ and $a_{2m+2}=1$. Meanwhile the same position bits in $b^{\prime }$ have similar values ( i.e., $b_{m+1}^{\prime }\equiv b_{2m+2}^{\prime }$). Hence, if $b_{m+1}^{\prime }\equiv b_{2m+2}^{\prime }=1$, it implies that $a_{m+1}$ is different than $b_{m+1}^{\prime }$. However, if $b_{m+1}^{\prime }\equiv b_{2m+2}^{\prime }=0$, it implies that $a_{2m+2}$ is different than $b_{2m+2}^{\prime }$. Thus, it is approved that in this case there must be at least a single bit that is different in **a** and $b^{\prime }$.

According to these possible cases, we conclude that **a**≠rotate(b,k),∀k∈(0,2m+1]. □

**Figure 5:**
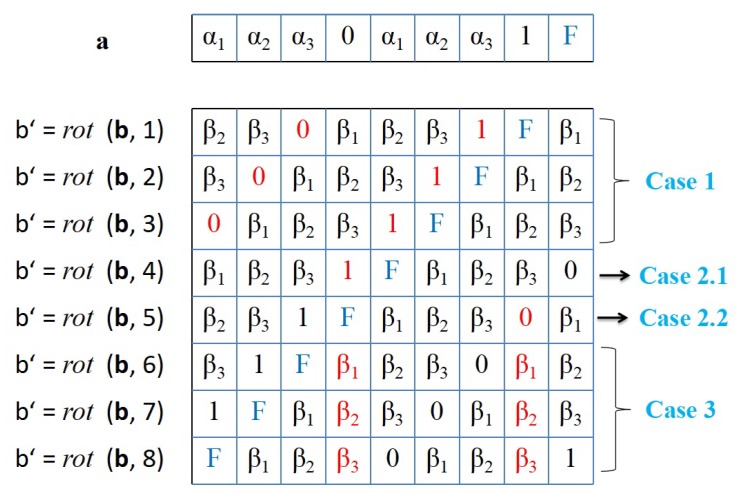
An illustrative example for the correctness of our cyclic unique seeds method when m=3.

Figure 5 illustrates an example for two binary representations of original lengths m=3 and which are transformed based on the proposed method to generated seeds with length (2m+3)=9 bits. The figure illustrate the cyclic uniqueness property between the expanded seeds for all the possible rotation cases (i.e., ∀k∈[1,8]). It is worthy noting that this cyclic uniqueness property holds even when the seed sequences are the same.

#### 5.1.2. Generating the Interleaved CH Sequence

This subsection explains how to generate the symmetric IQSF-CH sequence for any SU, say SU${}_{i}$. Suppose the number of channels for SU${}_{i}$ is n${}_{i}$ and the available channel set $A_{i}=\left\{A_{i}^{1},A_{i}^{2},\ldots ,A_{i}^{n_{i}}\right\}\subseteq L$. Let α be the *m*-bits binary representation of the selected seed channel $A_{i}^{seed}$ in $A_{i}$, which is transformed based on the above seed generation method into (2m+3)-bits seed sequence $a\overset{def}{=}\alpha ∥0∥\alpha ∥1∥F$. The generating steps for the IQSF-CH sequence of SU${}_{i}$ are as follows:

i Find the *hopping offset set* which contains the numbers in $\left[1,n_{i}\right]$ that are co-primes with $n_{i}$.
ii Find $p_{i}$ as the smallest prime which is not smaller than $n_{i}$.
iii Define an empty matrix M${}_{i}$ which has 2m+3 columns and $n_{i}\times p_{i}$ rows.
iv Fill M${}_{i}$ by mapping each bit in the seed **a** to a certain column as described below:
    1: **for**c=1:(2m+3)**do**
    2:  Select an *h-offset* randomly from the *offset set*.
    3:  **if** ($a_{c}==1$) **then**
    4:   Invoke (Algorithm 1) in Section 4.1 with $A_{i}$ and *h-offset* to generate an Q-CH sequence $\omega_{c}$.
    5:   Map the $c_{th}$ column of the matrix M${}_{i}$ with $\omega_{c}$.
    6:  **else**
    7:   **if** $\left(a_{c}==0\right)$ **then**
    8:    Invoke (Algorithm 2) in Section 4.1 with $A_{i}$ and *h-offset* to generate an S-CH sequence $\gamma_{c}$.
    9:    Map the $c_{th}$ column of the matrix M${}_{i}$ with $\gamma_{c}$.
  10:   **else** {($a_{c}==F$).}
  11:    Fill the $c_{th}$ column of matrix M${}_{i}$ with $A_{i}^{seed}$.
  12:   **end if**
  13:  **end if**
  14: **end for**
v Generate the IQSF-CH sequence by concatenating the matrix rows. The SU${}_{i}$ keep hopping according to this generated sequence and repeat it to rendezvous with its intended partner.

**Figure 6:**
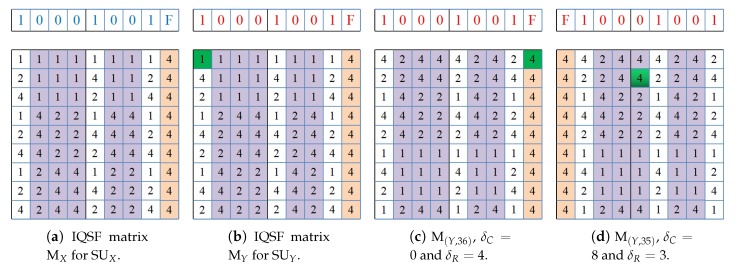
The constructed IQSF-CH matrices M${}_{X}$ for SU${}_{X}$ and M${}_{Y}$ for SU${}_{Y}$ and their rendezvous under the synchronous case as well as under the asynchronous case when M${}_{Y}$ is circularly rotated with (**c**) δ=(2⌈log${}_{2}5\rceil +3)\times 4+0=36$ time slots. (**d**) δ=(2⌈log${}_{2}5\rceil +3)\times 3+8=35$ slots.

For example, Figure 6a illustrates the IQSF-CH sequence construction matrix for SU${}_{X}$ which has the channel set $A_{X}=\left\{1,2,4\right\}$ that are available among the L=5 channels in the network. Without loss of generality, suppose that SU${}_{X}$ selects randomly the last channel in its ACS as its seed channel (i.e., $A_{X}^{seed}=4$). Hence, the binary representation of the selected channel (i.e., channel 4) is α=[100] which has a length of m=3-bits since ⌈log${}_{2}5\rceil =3$. This binary representation is then expanded to generate the seed a=[10001001F] for the SU${}_{X}$. The constructed IQSF matrix M${}_{X}$ has n×p=(3×3)=9 rows and 2m+3=9 columns. The SU fills its matrix columns with Q-CH or S-CH based on the corresponding bits of the seed sequence. On the other hand, suppose there is another SU (SU${}_{Y}$) which has identical ACS as SU${}_{X}$ and which select similar seed channel (i.e., $A_{X}^{seed}=A_{X}^{seed}=4$). The SU${}_{Y}$ constructs its IQSF-CH matrix M${}_{Y}$ in the same way as SU${}_{X}$ as illustrated in Figure 6b. However, the order of channels in each column sequence is determined based on the randomly selected hopping offsets. For instance, SU${}_{X}$ fill the first column in its IQSF matrix $M_{X}$ with a Q-CH sequence that is generated by invoking (Algorithm 1) with $A_{X}$ and *h-offset*=1. Meanwhile, SU${}_{Y}$ invokes (Algorithm 1) with $A_{Y}$ and *h-offset* = 2 to generates the Q-CH sequence assigned to its matrix first column. Under the asynchronous scenario, Figure 6c,d illustrates the shifted IQSF-CH matrix for SU${}_{Y}$ when it starts its channel hopping earlier than SU${}_{X}$ with 36 and 44 time slots, respectively. As can be seen, the rendezvous between the constructed IQSF-CH sequences of SU${}_{X}$ and SU${}_{Y}$ under these asynchronous misalignment cases is still achieved as indicated by the green entries at M${}_{Y}$ in Figure 6c,d.

### 5.2. Scheme Analysis

In this subsection, the theoretical performance of IQSF-CH is studied where the upper-bounds of MTTR under both channel availability models are derived.

**Theorem** **4.** *Under the symmetric channel availability model, the MTTR of IQSF-CH scheme is upper-bounded by (2⌈log${}_{2}L\rceil +3)\left(2p_{i}-1\right)$.*

**Proof** **of** **Theorem** **4.** To prove that IQSF-CH has MTTR ≤(2⌈log${}_{2}L\rceil +3)\left(2p_{i}-1\right)$, it is sufficient to prove that any arbitrary pair of IQSF-CH sequences, e.g., IQSF${}_{i}$ and IQSF${}_{j}$, can achieve a successful channel rendezvous within (2⌈log${}_{2}L\rceil +3)\left(2p_{i}-1\right)$ time slots. Let IQSF${}_{i}$ and IQSF${}_{j}$ be the corresponding IQSF-CH sequences of IQSF matrices M${}_{i}$ and M${}_{j}$ (i.e., IQSF${}_{i}$ and IQSF${}_{j}$ are generated by concatenating the rows of matrices M${}_{i}$ and M${}_{j}$, respectively). Without loss of generality, assume that SU${}_{j}$ starts its CH sequence δ time slots earlier than the start of SU${}_{i}$. This mean that in each CH period of SU${}_{i}$, the CH sequence of SU${}_{j}$ is *rotate*( IQSF${}_{j}$, δ). Clearly, the rendezvous between this pair of IQSF-CH sequences is the same as the rendezvous of their matrices M${}_{i}$ and M${}_{\left(j,\delta \right)}$. The matrices rendezvous is achieved when the same position entries in both matrices (M${}_{i}$ and M${}_{\left(j,\delta \right)}$) have the same channel index. Without loss of generality, Suppose that the time slot shift δ=(2⌈log${}_{2}L\rceil +3)\times \delta_{R}+\delta_{C}$, where $\delta_{R}\in \left[0,n_{j}p_{j}-1\right]$ denotes the shift of row and $\delta_{C}\in [0,(2\lceil$log${}_{2}L\rceil +2)]$ denotes the shift of column. Below, we consider the two different cases for rendezvous between IQSF${}_{i}$ and IQSF${}_{j}$.

***Case 1:***$\delta_{C}=0.$ This case implies that there is no column shift and only the row shift $\delta_{R}$ is exist. In the following, we consider the two sub-cases which happen according to whether the randomly selected seed channels by SU${}_{i}$ and SU${}_{j}$ are similar or different.

***Case 1.1:***$A_{i}^{seed}\equiv A_{j}^{seed}$: This case implies that the selected seed channels by SU${}_{i}$ and SU${}_{j}$ are the same which results in generating a similar seed sequence. In addition, the last columns in both matrices (i.e., F-type) are filled entirely with the same channel (i.e., $A_{i}^{seed}$). So, for any value of the row shift $\delta_{R}$, M${}_{i}$ and M${}_{\left(j,\delta \right)}$ are guaranteed to rendezvous no later than (2⌈log${}_{2}L\rceil +3)$ (at the first entry of last columns). For instance, refer to the previous example in Figure 6 which show the rendezvous of M${}_{X}$ and M${}_{Y}$. Under the synchronous case (i.e., when δ=0), the first channel rendezvous occurs in the first slot which is indicated by the green entry in M${}_{Y}$ (Figure 6b). However, Figure 6c shows the IQSF matrix of SU${}_{Y}$ when it is shifted with row shift $\delta_{R}=4$. This means that δ=(2⌈log${}_{2}5\rceil +3)\times 4+0=36$. The rendezvous between M${}_{X}$ and M${}_{\left(Y,36\right)}$ is achieved at their last columns.

***Case 1.2:***$A_{i}^{seed}≢A_{j}^{seed}$: This case implies that the seed sequences generated by the SUs are different and the F-type column in each matrix is filled with a different channel. For this sub-case, As recall that the columns types of the matrices are assigned based on these distinct seeds. Then, for any value of $\delta_{R}$, it is guaranteed that there must exist at least a single common column *d* in both matrices which is a Q-column in one matrix and a S-column in the other matrix, or visa-versa. In other words, the $d_{th}$ column of M${}_{i}$ (i.e., **a**${}_{d}$) contains a Q-CH sequence (reps., S-CH) while the same index $d_{th}$ column in M${}_{\left(j,\delta \right)}$ (i.e., **b**${}_{d}$) contains a S-CH (reps., Q-CH). By Theorem 1, it is known that the when SU${}_{i}$ hops on its channels according to a Q-CH (reps., S-CH) sequence and SU${}_{j}$ hops on its channels according to a S-CH (reps., Q-CH) sequence, they must rendezvous on a commonly available channel within at most $2p_{i}-1$ slots. However, since the elements of any specific column of each matrix appear in the corresponding IQSF-CH sequence every (2⌈log${}_{2}L\rceil +3)$ slots, $2p_{i}-1$ is multiplied by (2⌈log${}_{2}L\rceil +3)$.

***Case 2:***$\delta_{C}\neq 0.$ This case implies that there is a column shift $\delta_{C}\in [1,(2\lceil$log${}_{2}L\rceil +2)]$ between the matrices. Let **a** and **b** be the the generated seed sequences of SU${}_{i}$ and SU${}_{j}$, respectively. By Lemma 1, it is approved that **a** and **b** are *cyclic rotationally distinct* one to the other regardless of the similarity or difference between $A_{i}^{seed}$ and $A_{j}^{seed}$. Thus, for any column shift $\delta_{C}\in [1,2\lceil$log${}_{2}L\rceil +2]$ of matrix M${}_{j}$, there must exist a column *d* in matrix M${}_{i}$ which has different type than the same index $d_{th}$ column in the shifted matrix M${}_{\left(j,\delta \right)}$. Accordingly, the rendezvous is guaranteed to occur at the $d_{th}$ columns in the matrices, despite any values of $\delta_{R}$ and $\delta_{C}$. Hence, the MTTR upper-bound for this case is similar as the MTTR for the sub-case (1.2). For example, Figure 6d shows the shifted IQSF matrix of SU${}_{Y}$ when the column shift $\delta_{C}=8$ and the row shift $\delta_{R}=3$, which results in δ=(2⌈log${}_{2}5\rceil +3)\times 3+8=35$ slots. The first rendezvous between M${}_{X}$ in Figure 6a and M${}_{\left(Y,35\right)}$ in Figure 6d is achieved at time slot 14 since the fifth column of M${}_{X}$ is a Q-type while the fifth column of M${}_{\left(Y,35\right)}$ is S-type.

Above all, it is approved that the MTTR of IQSF-CH under the symmetric channel availability model is upper-bounded by (2⌈log${}_{2}L\rceil +3)\left(2p_{i}-1\right)$. □

**Theorem** **5.** *Under the asymmetric channel availability model, the MTTR between a pair of SUs (SU${}_{i}$ and SU${}_{j}$) performing the IQSF-CH scheme is upper-bounded by (2⌈log${}_{2}L\rceil +3)\times F\left(p_{i},p_{j}\right)$ where $F(p_{i},p_{j})$ is given by:*

(3) $$F\left(p_{i},p_{j}\right)=\left\{\begin{array}{cc} max\{\left(n_{j}-G\right)p_{j}+2p_{i}-1,\left(n_{i}p_{i}-Gp_{i}+1\right)p_{j}\} & if\;p_{i}<p_{j}\\ \left(max\left\{n_{i},n_{j}\right\}-G+1\right)p_{j} & if\;p_{i}=p_{j}\\ max\{\left(n_{i}-G\right)p_{i}+2p_{j}-1,\left(n_{j}p_{j}-Gp_{j}+1\right)p_{i}\} & if\;p_{i}>p_{j} \end{array}\right.$$

**Proof** **of** **Theorem** **5.** Similar to the analysis procedure of the symmetric model, the guaranteed rendezvous between any arbitrary pair of IQSF-CH sequences (IQSF${}_{i}$ and IQSF${}_{j}$) under the asymmetric channel availability model can be approved by proving the deterministic rendezvous of their IQSF matrices despite any misalignment of their start times. Specifically, the rendezvous between M${}_{i}$ and M${}_{\left(j,\delta \right)}$ is proved to be deterministic within a bounded time despite any shift δ∈(2⌈log${}_{2}L\rceil +3)\times \delta_{R}+\delta_{C}$ in M${}_{\left(j,\delta \right)}$. However, as the ACSs ($A_{i}$ and $A_{j}$) in this model are diverse, we analyze the cases which consider the different relations of $p_{i}$ and $p_{j}$ as well as the channels used for seeds generation in deriving the MTTR upper-bound of this model.

***Case 1:***$\delta_{C}=0.$ This case implies that there is no column shift. So, The MTTR upper-bound is derived by considering the similarity or difference between the randomly selected seed channels.

***Case 1.1:***$A_{i}^{seed}\equiv A_{j}^{seed}$: This sub-case implies that the generated seed sequences and the F-type columns for both SUs are similar. Therefore, for this sub-case, it can be easily approved in a similar way as the proof of sub-case 1.1 in Theorem 4 that within the upper-bound (2⌈log${}_{2}L\rceil +3)$, M${}_{i}$ and M${}_{\left(j,\delta \right)}$ can rendezvous despite any values of $\delta_{R}$.

***Case 1.2:***$A_{i}^{seed}≢A_{j}^{seed}$. This case implies that the F-type columns and the seed sequences are distinct. According to that and sine the column types of the matrices are assigned based on distinct seeds, it is guaranteed that there must exist at least a single common column *d* in both matrices which is a Q-column in one matrix and a S-column in the other matrix, and visa-versa, despite any value of $\delta_{R}$. Recall the proof of Theorem 2; it was approved that when SUs hop on their channels according to the different asymmetric-role CH sequences (Q-CH versus S-CH ), they must rendezvous on a commonly available channel within a bounded MTTR. The MTTR upper-bound which is given by Equation (1) depends on the number of available channels and the corresponding prime numbers ( i.e., $n_{i},n_{j},p_{i},p_{j}$) as well as on *G*. However, as the elements of any column in each matrix appear in the corresponding IQSF-CH sequence every (2⌈log${}_{2}L\rceil +3)$ slots, the MTTR upper-bound of the asymmetric-role approach in Equation (1) is multiplied by (2⌈log${}_{2}L\rceil +3)$. In particular, suppose that **a**${}_{d}=1$ and **b**${}_{d}=0$ which indicates that the $d_{th}$ column in M${}_{i}$ contains a Q-CH sequence while the $d_{th}$ column in M${}_{\left(j,\delta \right)}$ contains a S-CH sequence. Accordingly, the rendezvous happen when the same position entries in these $d_{th}$ columns contain the same channel and the MTTR upper-bound is given by:

(4) $$MTTR\leq \left\{\begin{array}{cc} \left(2\left\lceil log_{2}L\right\rceil +3\right)\times \left(n_{j}-G\right)p_{j}+2p_{i}-1 & \mathrm{if}\;p_{i}<p_{j}\\ \left(2\left\lceil log_{2}L\right\rceil +3\right)\times \left(n_{j}-G+1\right)p_{j} & \mathrm{if}\;p_{i}=p_{j}\\ \left(2\left\lceil log_{2}L\right\rceil +3\right)\times \left(n_{j}p_{j}-Gp_{j}+1\right)p_{i} & \mathrm{if}\;p_{i}>p_{j} \end{array}\right.$$

On the other hand, when **a**${}_{d}=0$ and **b**${}_{d}=1$ which indicates that the $d_{th}$ columns in M${}_{i}$ and M${}_{\left(j,\delta \right)}$ contain S-CH and Q-CH sequences, respectively, the MTTR upper-bound is given by:

(5) $$MTTR\leq \left\{\begin{array}{cc} \left(2\left\lceil log_{2}L\right\rceil +3\right)\times \left(n_{i}p_{i}-Gp_{i}+1\right)p_{j} & \mathrm{if}\;p_{i}<p_{j}\\ \left(2\left\lceil log_{2}L\right\rceil +3\right)\times \left(n_{i}-G+1\right)p_{i} & \mathrm{if}\;p_{i}=p_{j}\\ \left(2\left\lceil log_{2}L\right\rceil +3\right)\times \left(n_{i}-G\right)p_{i}+2p_{j}-1 & \mathrm{if}\;p_{i}>p_{j} \end{array}\right.$$

By generalization the similar terms in Equations (4) and (5) to obtain unified formulas for them, the MTTR upper-bound can be represented as:

(6) $$MTTR\leq \left\{\begin{array}{cc} \left(2\left\lceil log_{2}L\right\rceil +3\right)\times max\left\{\left(n_{j}-G\right)p_{j}+2p_{i}-1,\left(n_{i}p_{i}-Gp_{i}+1\right)p_{j}\right\} & \mathrm{if}\;p_{i}<p_{j}\\ \left(2\left\lceil log_{2}L\right\rceil +3\right)\times \left(max\left\{n_{i},n_{j}\right\}-G+1\right)p_{j} & \mathrm{if}\;p_{i}=p_{j}\\ \left(2\left\lceil log_{2}L\right\rceil +3\right)\times max\left\{\left(n_{i}-G\right)p_{i}+2p_{j}-1,\left(n_{j}p_{j}-Gp_{j}+1\right)p_{i}\right\} & \mathrm{if}\;p_{i}>p_{j} \end{array}\right.$$

So, for this sub-case, it is approved that M${}_{i}$ and M${}_{\left(j,\delta \right)}$ are guaranteed to rendezvous at their $d_{th}$ columns despite any row shift $\delta_{R}$ value within a period of (2⌈log${}_{2}L\rceil +3)\times F\left(p_{i},p_{j}\right)$ time slots where $F(p_{i},p_{j})$ depends on the relation between $p_{i}$ and $p_{j}$ as shown in Equation (3). It is worthy noting that when ($p_{i}\neq p_{j}$), the matrices rendezvous can also be achieved even when **a**${}_{d}=$**b**${}_{d}=1$ which indicates that both the $d_{th}$ columns in M${}_{i}$ and M${}_{\left(j,\delta \right)}$ contain Q-CH sequences. This is because $p_{i}$ and $p_{j}$ are co-prime if they are not equal. Hence, it can be proved easily with the help of Chinese Reminder theorem that matrices will rendezvous not later than (2⌈log${}_{2}L\rceil +3)\times p_{i}p_{j}$.

**Figure 7:**
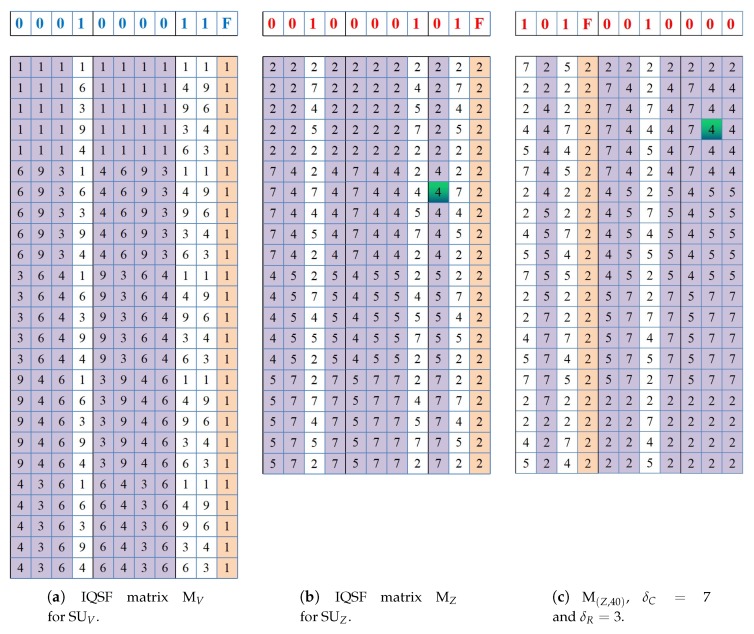
The constructed IQSF matrices for SU${}_{V}$ and SU${}_{Z}$ when L=10 and their rendezvous. (**a**) SU${}_{V}$ selects $A_{V}^{seed}=1$, hence, $\alpha_{V}=\left[0001\right]$ and the seed of SU${}_{V}$ is **a**${}_{V}=\left[0001000011F\right]$. (**b**) SU${}_{Z}$ selects $A_{Z}^{seed}=2$, hence, $\beta_{Z}=\left[0100\right]$ and the seed of SU${}_{Z}$ is **b**${}_{Z}=\left[0010000101F\right]$. (**c**) the IQSF matrix M${}_{Z}$ for SU${}_{Z}$ when it is circularly rotated with δ=(2⌈log${}_{2}10\rceil +3)\times 3+7=40$ slots.

To illustrate the guaranteed rendezvous of IQSF-CH when SUs has distinct ACSs, consider the previous example of the two asymmetric-role SUs (sender SU${}_{V}$ and receiver SU${}_{Z}$) in Figure 1 at Section 4. Suppose that there is L=10 global channels in the network. Figure 7 shows their constructed IQSF matrices when they perform CH without the assumption of the pre-assigned role (i.e., symmetric approach). As the SUs have different number of available channels, their IQSF constructed matrices have different sizes. The matrix M${}_{V}$ for SU${}_{V}$ has (2⌈log${}_{2}10\rceil +3)=11$ columns and $n_{v}p_{v}=\left(5\times 5\right)=25$ rows while M${}_{Z}$ for SU${}_{Z}$ has 11 columns and $n_{v}\times 4_{v}=\left(5\times 5\right)=20$ rows. As shown in the figure, rendezvous of M${}_{V}$ and M${}_{Z}$ for the synchronous case (i.e., when δ=0) occurs between the ninth columns of M${}_{V}$ and M${}_{Z}$ which is indicated by the green entry in M${}_{Z}$.

***Case 2:***$\delta_{C}\neq 0.$ In this case, it holds that there is a column shift in M${}_{\left(j,\delta \right)}$. Similar to the proof of Case 2 in Theorem 4, it can be easily verified based on Lemma 1 that the seed sequences **a** and **b** are *cyclic rotationally distinct* one to the other despite any column shift. This cyclic uniqueness feature holds even when the seeds are similar. Accordingly, the existence of different type columns in matrices is guaranteed at least once which consequently results in a guaranteed rendezvous despite any value of the row shift $\delta_{R}$. The MTTR upper-bound for this case is similar as the bound in Equation (6). For example, Figure 7c shows the shifted IQSF matrix of SU${}_{Z}$ when the column shift $\delta_{C}=7$ and the row shift $\delta_{R}=3$, which results in δ=(2⌈log${}_{2}10\rceil +3)\times 3)+7=40$ slots. The first rendezvous between M${}_{V}$ in Figure 7a and M${}_{\left(Z,40\right)}$ in Figure 7c is achieved between their tenth columns.

To sum up, it is proved that the MTTR upper bound when SUs have diverse ACSs depends mainly on $p_{i}$ and $p_{j}$ as shown by $F(p_{i},p_{j})$ in Equation (3) while impacted by (2⌈log${}_{2}L\rceil +3)$. □

**Theorem** **6.** *The IQSF-CH scheme can provide a full RD.*

**Proof** **of** **Theorem** **6.** The proof is given in Appendix B. □

## 6. Performance Evaluation

This section presents the performance evaluation of the developed QS-CH and IQSF-CH schemes as compared with some extant blind CH rendezvous schemes. The proposed QS-CH scheme is compared with six representative asymmetric-role schemes (four GC schemes, FDCH-RP [27], PCH [28], AAsync [31], and FARCH [30,34] as well as two LC schemes named as D-QCH in [32] and SJRW [23]). On the other side, the proposed IQSF-CH scheme is compared with six representative symmetric-role CH schemes (four GC schemes, EJS [20], T-CH [40], D-CH [40], and S-QCH in [32] as well as two LC schemes named as ZOS [44] and MTP [21,43]).

These CH schemes are selected for comparison due to their deterministic and extant performance in providing asynchronous blind pairwise rendezvous in CRNs. Furthermore, all of the symmetric-role compared schemes do not relay on any unfavorable assumption such as the utilizing of SU’s IDs to guide rendezvous. As an exception, the ID-based D-CH scheme in [40] is selected due to its matrix-based design which makes it suitable for the comparison with our IQSF-CH scheme. It is worth noting that even though SSS [22] is a recent LC-based scheme, it is not included in the simulation since it does not guarantee rendezvous under some scenarios as explained in Section 2.2.

### 6.1. Simulation Setup

Extensive simulations using MATLAB have been conducted to compute the ETTR and MTTR required by the compared schemes to achieve rendezvous between a pair of SUs (a sender SU${}_{i}$ and a receiver SU${}_{j}$). The simulations are performed under asynchronous environments in which the clock drift δ between SUs is selected randomly in each run. The schemes are simulated under the asymmetric channel availability model in which SUs have different ACSs, but there exist some commonly available channels in order to realize rendezvous. The *G* number of common channels is determined based on the following conditions: $\left(1\leq G\leq min\left\{n_{i},n_{j}\right\}\right)$ and $\left(n_{i}+n_{j}-G\leq L\right)$. In the simulation, a parameter σ(0<σ≤1) is introduced to adjust the ratio of the number of local available channels to that of global channels (i.e., $\sigma_{i}=\frac{n_{i}}{L}$ and $\sigma_{j}=\frac{n_{j}}{L}$). The $n_{i}=\sigma_{i}L$ and $n_{j}=\sigma_{j}L$ available channels of the SUs are selected randomly from the GCS in the different runs. As the D-CH scheme rely on the SU’s IDs, it is simulated when each SU has 7-bit ID sequence. The schemes are simulated under various settings of the $(L,n_{i},n_{j}$ and G) parameters as described in Table 3. These settings are as follows: (i) Vary the value of *L* and fix the ratios of SUs available channels ($\sigma_{i},\sigma_{j}$) as well as the number of commonly available channels *G*; (ii) Fix the values of *L* and ($\sigma_{i},\sigma_{j}$) while varying the value of *G*; (iii) Fix the values of *L* and *G* while varying the values of ($\sigma_{i},\sigma_{j}$).

For each value of the simulation parameters, the results are obtained as the expected and maximum values of TTR for more than $10^{5}$ independent runs.

### 6.2. Influence of the Number of Licensed Channels (L)

In this simulation, the value of *L* is varied from 10 to 60 with steps of 5 while the values of $\sigma_{i},\sigma_{j},$ and *G* are set as ($\sigma_{i}=0.2,\sigma_{j}=0.3$ and G=0.1). However, since the AAsync and T-CH schemes require *L* and L+1, respectively to be primes, we simulate them under the closest values to *L* which satisfy their restrictions for fairness. Furthermore, it is found through simulations that the primitive roots which are valid to construct the default and elementary sequences in AAsync can not be obtained for some primes. Hence, the AAsync scheme was simulated only under the valid *L* values.

**Figure 8:**
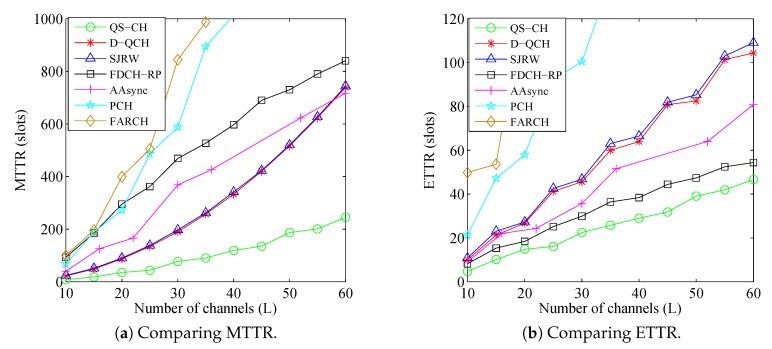
Comparison results of the asymmetric-role schemes when ($n_{i}=0.2,n_{j}=0.3,$ and G=0.1).

**Figure 9:**
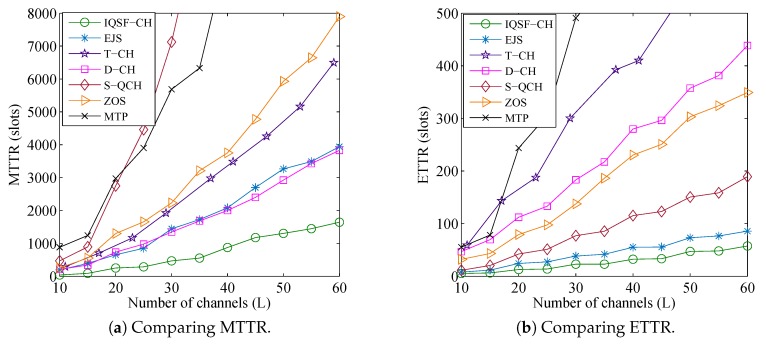
Comparison results of the symmetric-role schemes when ($n_{i}=0.2,n_{j}=0.3,$ and G=0.1).

Figure 8 and Figure 9 show the comparisons results under such parameters for the asymmetric-role and symmetric-role schemes, respectively. They are evident that our schemes can achieve faster rendezvous. The figures also show that the TTR results of all schemes increase relatively with the increase of *L*. However, due to the efficient LC-based design of our schemes where CH-sequences are generated based on the unrestricted ACSs only, the increase in their TTR results are slower than others.

Figure 8 illustrates the superior performance of the proposed QS-CH as compared to the other asymmetric-role schemes where it can achieve up to 68% and 36% improvement in MTTR and ETTR, respectively. Although D-QCH and SJRW are LC schemes, Figure 8 shows that they consume long MTTR and ETTR. This is mainly due to the long periods by which their receiver stay on each available channel which prolongs their TTRs specially when the receiver first stay channels are not common with its sender. On the other hand, the MTTRs of the other GC schemes (i.e., FDCH-RP, AAsync, PCH, and FARCH) are long since their CH sequences are constructed based on the whole GCS which enlarge their CH periods. However, since FARCH does not adopt any strategy for replacing the unavailable channels and due to the large sequences of the PCH, they produces much longer TTR results than others where some of the values are not displayed in the figures. It is also noticed that while FDCH-RP consumes long MTTR (Figure 8a), it shows its superiority in the sense that its ETTR is shorter than D-QCH, SJRW, and AAsync (Figure 8b). The reason is that FDCH-RP randomly replace the unavailable channels in its CH sequence with available ones which boost its ETTR performance.

As for the symmetric-role schemes, Figure 9 shows that our IQSF-CH provides the best results where it can reduce the MTTR and ETTR up to 73% and 50% as compared to the closest schemes. Even though MTP and ZOS are LC-based schemes similar to IQSF-CH, they provide much longer TTR results where some of the MTP values are not displayed. This is mainly due to their inefficient designs where they generate very large LC-based CH sequences. On the other hand, the T-CH scheme produces longer TTR results than EJS and D-CH although it has shorter CH period than them. The reason is that T-CH attempts rendezvous on all the licensed channels (i.e., even those which are unavailable). The MTTR of S-QCH is much longer others where some of its values are not displayed in the figures. This is mainly due to its longer CH sequences which increase drastically with the increase of *L*. However, its ETTR is much shorter than other schemes except our IQSF-CH and EJS, which indicates that its worst case TTRs are happening rarely.

### 6.3. Influence of the Number of Common Channels (G)

**Figure 10:**
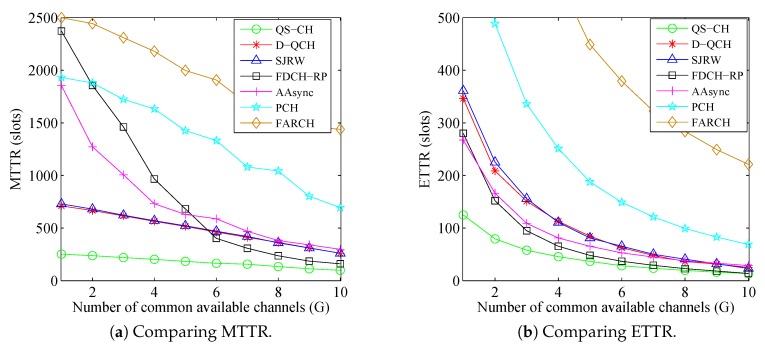
Comparison results of the asymmetric-role schemes when ($L=50,n_{i}=10,n_{j}=15$) under different values of *G*.

**Figure 11:**
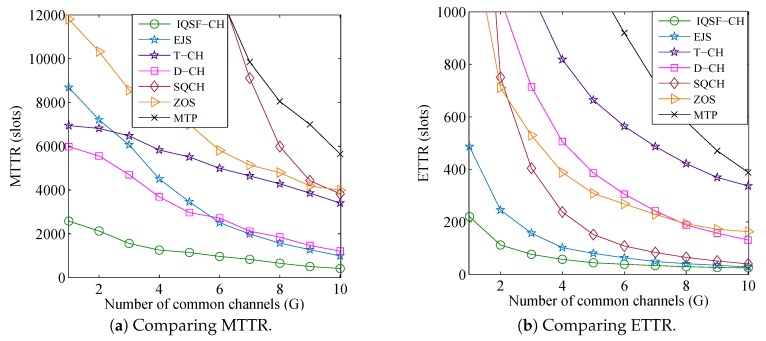
Comparison results of the symmetric-role schemes when ($L=50,n_{i}=10,n_{j}=15$) under different values of *G*.

In this simulation, the number of licensed channels is fixed as L=50 and the available channel ratios are set as ($\sigma_{i}=0.2$ and $\sigma_{i}=0.3$) which indicates that $n_{i}=0.2\times 50=10$ and $n_{j}=0.3\times 50=15$. Meanwhile, *G* is varied from 1 to 10. Figure 10 and Figure 11 show the comparisons results under such parameters for the asymmetric-role and symmetric-role schemes, respectively. The figures show that the TTR results of all schemes decrease with the increase of *G*. This is because SUs have more chances to rendezvous when there is a large number of common channels between their ACSs.

As for the asymmetric-role schemes, Figure 10 illustrates the superior performance of the proposed QS-CH as compared to the other schemes specially when *G* is small. It shows that QS-CH has at least 64% and 30% improvement in MTTR and ETTR, respectively when (G≤4). The figure also shows that when G>5, the MTTR of FDCH-RP is shorter than those of (D-QCH and SJRW) and is getting closer to the QS-CH’s MTTR. However, its MTTR is very long when *G* is smaller than 5. The results for the PCH and FARCH are still much longer than others.

As for the symmetric-role schemes, Figure 11 shows that the proposed IQSF-CH scheme can achieve faster rendezvous than the other schemes. Figure 11a,b illustrates that IQSF-CH can provide at least 59% and 43% reduction in MTTR and ETTR, respectively, when (G≤5). Since the SUs in the T-CH scheme are accessing all the channels even those which are unavailable, its MTTR is slowly decreasing with the increase of *G*. On the contrary, as EJS and D-CH replace the unavailable channels in their CH sequences with available ones, their downward trend is faster than it is in the T-CH scheme. The MTTR for the S-QCH and MTP are still much longer than others where some of their MTTR values are not displayed in the figures. However, S-QCH ETTR is shorter than D-CH and ZOS when G>3.

### 6.4. Influence of Large Number of Available Channels $\left(n_{i},n_{j}\right)$

**Figure 12:**
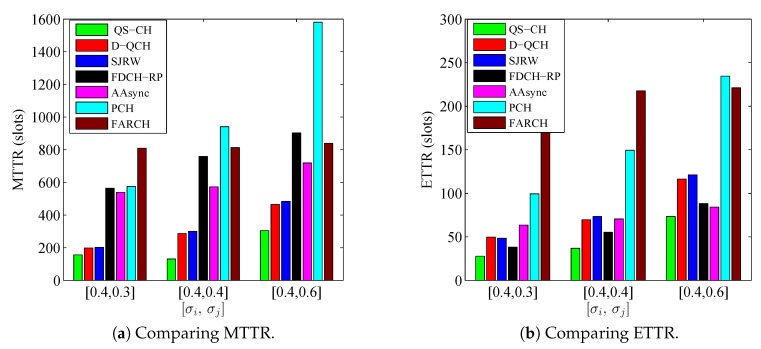
Comparison results of the asymmetric-role schemes under different combinations of $\left[n_{i},n_{j}\right]$ when (L=30 and G=3) .

**Figure 13:**
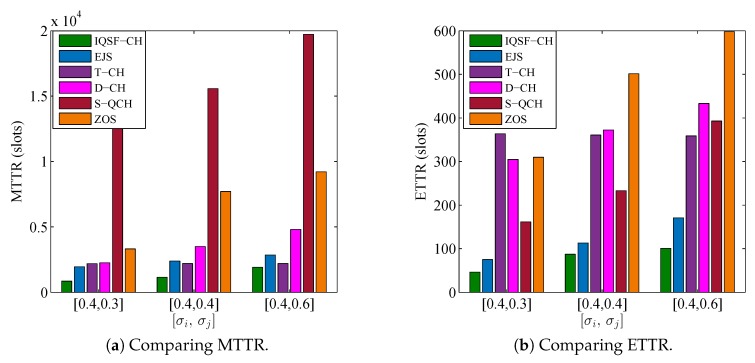
Comparison results of the symmetric-role schemes when (L=30 and G=3) under different combinations of $\left[n_{i},n_{j}\right]$.

The simulation setting in this subsection is conducted to compare the schemes’ performance when SUs have large number of available channels. In this simulation, the numbers of licensed and common channels are fixed as L=30 and G=0.1×L=3, respectively. Meanwhile, the SUs are simulated under different combinations of their local available channels. In particular, $\sigma_{i}$ is set to 0.4 while the values of $\sigma_{j}$ are {0.3,0.4,0.6}. Figure 12 and Figure 13 depict the comparisons results under such parameters for the asymmetric-role and symmetric-role schemes, respectively. The figures are evidence that our schemes still out-perform the other ones significantly even when $n_{i}$ and $n_{j}$ are large.

In Figure 12, it is shown that the QS-CH scheme performance always out-performs the other schemes under all combinations. Although the MTTRs of D-QCH and SJRW are the closest to the QS-CH’s MTTR under the [0.4,0.3] combination, QS-CH still outperforms them significantly in terms of MTTR under the other combinations as well as in terms of ETTR.

On the other side, Figure 13 depicts the comparison results for the symmetric-role schemes except MTP which is omitted because of its extremely longer results. It is evident that IQSF-CH can obtain the best performance under all the combinations of $\left[n_{i},n_{j}\right]$. It also shown in (Figure 13a) that when $\left[\sigma_{i}=0.4,\sigma_{j}=0.6\right]$ which indicates that $\left[n_{i},n_{j}\right]=\left[12,18\right]$, the MTTR of T-CH is better than EJS and relatively close to IQSF-CH. However, the ETTR of T-CH is still very long as shown in (Figure 13b).

## 7. Conclusions

In this paper, we have proposed two matrix-based asymmetric and symmetric CH schemes, named as QS-CH and IQSF-CH, for achieving blind rendezvous in distributed CRNs. The proposed schemes only utilize the unrestricted local ACSs to generate their CH sequences without relaying on any extra expenditure such as the SUs’s IDs, which is more favorable in heterogeneously distributed environments. Theoretical analyses were carried out to prove the deterministic rendezvous of our schemes. It was proven that they can provide rendezvous within a short MTTR whose upper-bound is completely irrelevant of the number of global licensed channels *L* in the QS-CH scheme while it is only impacted by an O(log${}_{2}L)$ factor in the IQSF-CH scheme. Furthermore, extensive simulations have been conducted to demonstrate the proposed schemes’ efficiency. The simulation results verify that they significantly outperform the previous works in terms of MTTR and ETTR.

## Figures and Tables

**Table 1:** Theoretical performance comparison for the asymmetric-role schemes.

Scheme	MTTR		Full	Class
	Symmetric Model	Asymmetric Model	RD	
ACH [25]	$L^{2}-L+1$	$L^{2}$	✓	GC
ARCH [26]	2L+1 ${}^{🟉}$	$L^{2}$ ${}^{🟉}$	✓ ${}^{🟉}$	GC
CSAC [24]	−	(i) $n_{r}p_{s}-G+1$, (when $n_{r}=kp_{S}$)	✓	LC
		(ii) $n_{r}^{2}\left(p_{s}-1\right)-\left(G-2\right)n_{r}$, (when $n_{r}\neq kp_{S}$)		
FDCH-RB [27]	(i) *L* ${}^{†}$, (when *L* is even)	(i) $L^{2}+L$, (when *L* is even)	✓	GC
	(ii) L−1 ${}^{†}$, (when *L* is odd)	(ii) $L^{2}-1$, (when *L* is odd)		
PCH [28]	2L−1	L(2L−1)	✓	GC
WFM [29]	L+1	$L^{2}$	✓	GC
D-QCH [32]	2L	$(n_{r}-G+1)L$	✓	LC
FARCH [30,34]	(i) L+1, (when *L* is even)	$L^{2}$	✓	GC
	(ii) *L*, (when *L* is odd)			
AAsync [31]	−	$L^{2}$ ${}^{♯}$	✓ ${}^{♯}$	GC
SJ-RW [23,33]	2L−1	$L^{2}$	✓	LC
QS-CH	$2p_{s}-1$	(i) $\left(n_{r}-G+1\right)p_{r}$, (when $p_{s}=p_{r}$)	✓	LC
		(ii) $\left(n_{r}-G\right)p_{r}+\left(2p_{s}-1\right)$, (when $p_{s}<p_{r}$)		
**[This paper]**		(iii) $\left(n_{r}p_{r}-Gpr+1\right)p_{s}$, (when $p_{s}>p_{r}$)		

**Remarks**: *L* is the number of global licensed channels; $n_{s}$ and $n_{r}$ are the number of available channels for the sender and receiver SUs, respectively; $p_{s}$ and $p_{r}$ are the smallest primes not smaller than $n_{s}$ and $n_{r}$, respectively; *G* is the number of commonly available channels between SUs; 🟉 denotes that the results are valid when *L* is even (refer to [26] for further details); † denotes that this bound is valid only when all channels are available; ♯ indicates that only when L+1 is a prime; − indicates that results are not given in the original papers.

**Table 2:** Theoretical performance comparison for the symmetric-role schemes.

Scheme	MTTR		No	Full	Class
	Symmetric Model	Asymmetric Model	ID	RD	
JS [35]	3P	3LP(P−G)+3P	✓	✓	GC
EJS [20]	4P	4P(P−G+1)	✓	✓	GC
S-ACH [17]	$6\lambda L^{2}$	$6\lambda L^{2}$	*X*	✓	GC
E-AHW [18]	3λP	3(λ+1)P(L−G+1)	*X*	✓	GC
DRDS [36]	3P	$3P^{2}+2P$	✓	✓	GC
FRCH [37]	2L+1	L(2L+1) ${}^{🟉}$	✓	*X*	GC
SSB [38]	2L−2	(L−1)(2L+1) ${}^{🟉}$	✓	*X*	GC
SARCH [26]	4L+2 ${}^{†}$	$8L^{2}+8L$ ${}^{†}$	✓	*X*	GC
HH [41]	−	$2P^{2}+2P$	✓	✓	LC
T-CH [40]	−	$2L^{2}+\left\lfloor \frac{L}{2}\right\rfloor \times L$ ${}^{‡}$	✓	✓	GC
D-CH [40]	−	$\left(\lambda +1\right)\left(L^{2}+L\right)$	*X*	✓	GC
CBH [19]	−	$2l_{p}\times max{\left\{p_{i},p_{j}\right\}}^{2}$	*X*	✓	LC
PDP [42]	−	L(2L+1)	✓	*X*	GC
L-PDP [42]	−	$T_{i}T_{j}-min\left\{T_{i},T_{j}\right\}G+1$ ${}^{*}$	✓	*X*	LC
MTP [21,43]	$2n_{i}^{2}\times 32\left(loglogL+1\right)$	$2{\left(max\left\{n_{i},n_{j}\right\}\right)}^{2}\times 32\left(loglogL+1\right)$	✓	✓	LC
ZOS [44]	−	(12⌈log${}_{2}L\rceil +2)\left(p_{j}p_{j}+max\left\{p_{i},p_{j}\right\}\right)$	✓	✓	LC
SSS [22]	$6p_{i}$ ${}^{⋄}$	(i) $6p_{i}^{2}$ ${}^{⋄}$, (when $p_{i}=p_{j}$ or $p_{i}⩾2p_{j}$)	✓	✓ ${}^{⋄}$	LC
		(ii) $6p_{i}^{2}p_{j}$, (when $p_{j}<p_{i}<2p_{j}$)			
S-QCH [32]	L(2L+1)	$\left(n_{j}-G+1\right)L\left(2L+1\right)$	✓	✓	GC
IQSF-CH	(2⌈log${}_{2}L\rceil +3)\left(2p_{i}-1\right)$	(i) (2⌈log${}_{2}L\rceil +3)\times \left(max\left\{n_{i},n_{j}\right\}-G+1\right)p_{j}$ (when $p_{i}=p_{j}$)	✓	✓	LC
**This paper**		(ii) $max\{\left(n_{j}-G\right)p_{j}+2p_{i},\left(n_{i}p_{i}-Gp_{i}\right)p_{j}\}$ ×(2⌈log${}_{2}L\rceil +3)$, (when $p_{i}\neq p_{j}$)			

**Remarks**: *P* is the smallest prime ⩾L; $n_{i}$ and $n_{j}$ are the number of available channels for the pair of SUs (SU${}_{i}$ and SU${}_{j}$), respectively; $p_{i}$ and $p_{j}$ are the smallest primes not smaller than $n_{i}$ and $n_{j}$, respectively; λ denotes the unique λ-bit ID; $l_{p}$ is a constant determined by SUs’ IDs (refer to [19] for details); $T_{i}$ and $T_{j}$ are the periods of the L-PDP CH sequences for SU${}_{i}$ and SU${}_{j}$; * denotes that the results are only valid when the great common divisor gcd($T_{i}$, $T_{j}$)=1; 🟉: denotes that the conclusion is only valid for some values of *L* (refer to [37] and [39] for further details); † denotes when 2L+1 is a prime and all channels are available. ‡ denotes when *L* is prime; ⋄ denotes that results are not valid under some scenarios (refer to [45] for details)

**Table 3:** Simulation parameters.

Parameter	Value		
	Setting I	Setting II	Setting III
Number of global licensed channels (L)	(10−60)	50	30
Number of local available channels for SU${}_{i}$ $\left(n_{i}\right)$	0.2L	10	0.4L
Number of local available channels for SU${}_{j}$ $\left(n_{j}\right)$	0.3L	15	(0.3L,0.4L,0.6L)
Number of commonly available channels (G)	0.1L	(1−10)	3

## Appendix A

Without loss of generality, suppose that the sender starts Q-CH later than its intended receiver which has only one common channel with sender. In the following sub-cases, we derive the theoretical MTTR upper-bound required for the sender to guarantee rendezvous starting from its first frame.

***Case 2.1:***$(p_{s}$ mod $n_{r}p_{r})<p_{r}$. This sub-case indicates that during each sender frame, the receiver sequence is deviated clockwise with amount less than the length of its stay frame. Accordingly, starting from its first frame, the sender needs to repeat its frame only for $\left\lceil \frac{p_{r}\left(n_{r}-1\right)}{p_{s}\mathrm{mod}\;n_{r}p_{r}}\right\rceil$ times in order to overlap with the whole elements in the receiver sequence. Hence, the rendezvous for this sub-case is guaranteed to occur no later than $\left(\left\lceil \frac{p_{r}\left(n_{r}-1\right)}{p_{s}\mathrm{mod}\;n_{r}p_{r}}\right\rceil +1\right)\times p_{s}$ time slots. For example, suppose that $n_{r}=p_{r}=3$ and $p_{s}=11$, the MTTR is $\left(\left\lceil \frac{3\times (3-1)}{11\;\mathrm{mod}\;9}\right\rceil +1\right)\times 11=\left(\left\lceil \frac{6}{2}\right\rceil +1\right)\times 11=44$ slots which is less than half of the loosely upper-bound $\left(n_{r}p_{r}\right)p_{s}=99$ slots. As ($p_{s}$ mod $n_{r}p_{r}$) is the denominator in the upper-bound formula, it is obvious that when it is closer to $p_{r}$, the MTTR is shorter.

***Case 2.2:*** ($p_{s}$ mod $n_{r}p_{r})>p_{r}\left(n_{r}-1\right)$. In this sub-case, the receiver sequence is deviated clockwise with amount that is $\left(n_{r}-1\right)$ times bigger than the length of its stay frame. However, since this deviation is less than $n_{r}p_{r}$, it implies that the receiver sequence is deviated with $p_{r}n_{r}-\left(p_{s}\mathrm{mod}\;n_{r}p_{r}\right)$ in the anticlockwise orientation. So, the sender need to repeat its frame only for $\left\lceil \frac{p_{r}\left(n_{r}-1\right)}{p_{r}n_{r}-\left(p_{s}\mathrm{mod}\;n_{r}p_{r}\right)}\right\rceil$ times in order to guarantee rendezvous with its receiver. Hence, the MTTR for this sub-case is $\leq \left(\left\lceil \frac{p_{r}\left(n_{r}-1\right)}{p_{r}n_{r}-\left(p_{s}\mathrm{mod}\;n_{r}p_{r}\right)}\right\rceil +1\right)\times p_{s}$. For example, if $p_{s}=7$ in the previous example, the MTTR is $\left(\left\lceil \frac{6}{9-(7\;\mathrm{mod}\;9)}\right\rceil +1\right)\times 7=28$ slots.

***Case 2.3:*** ($p_{s}$ mod $n_{r}p_{r})=\left\lceil \frac{p_{r}n_{r}-1}{i}\right\rceil$ or $p_{r}n_{r}-\left\lceil \frac{p_{r}n_{r}-1}{i}\right\rceil ,i\in \left[2,p_{r}-1\right]$. In this sub-case, the receiver sequence is deviated with amount that is $\approx \frac{1}{i}$ of its length. The deviation is in a clockwise orientation when ($p_{s}$ mod $n_{r}p_{r})=\left\lceil \frac{p_{r}n_{r}-1}{i}\right\rceil$ or in anticlockwise when it equals to $p_{r}n_{r}-\left\lceil \frac{p_{r}n_{r}-1}{i}\right\rceil$. Hence, the MTTR for SUs rendezvous $\leq \left[\left(p_{r}-i+1\right)\left(n_{r}-1\right)\right]p_{s}$.

***Case 2.4:*** ($p_{s}$ mod $n_{r}p_{r})=p_{r}+i$ or $p_{r}\left(n_{r}-1\right)-i,i$ is even number $\in [2,\left\lfloor \frac{p_{r}}{2}\right\rfloor ]$. In this sub-case, MTTR $\leq \left[(2p_{r}-2i)\right]p_{s}$.

***Case 2.5:*** ($p_{s}$ mod $n_{r}p_{r})=p_{r}\left\lfloor \frac{p_{r}}{i}\right\rfloor +1$ or $p_{r}n_{r}-\left(p_{r}\left\lfloor \frac{p_{r}}{i}\right\rfloor +1\right),i\in \left[2,\left\lfloor \frac{p_{r}}{2}\right\rfloor \right]$. In this sub-case, MTTR $\leq \left(p_{r}+\left(i-1\right)\right)p_{s}$.

***Case 2.6:*** ($p_{s}$ mod $n_{r}p_{r})=p_{r}\left\lfloor \frac{p_{r}}{i}\right\rfloor -1$ or $p_{r}n_{r}-\left(p_{r}\left\lfloor \frac{p_{r}}{i}\right\rfloor -1\right),i\in \left[2,\left\lfloor \frac{p_{r}}{2}\right\rfloor \right]$. In this sub-case, MTTR $\leq \left(p_{r}+\left\lceil \frac{p_{r}}{i}\right\rceil \right)p_{s}$.

From the above sub-cases, it is obvious that the MTTR is much less than $p_{s}\left(p_{r}n_{r}\right)$ even when G=1. However, due to the difference in these sub-cases formulas, the MTTR upper-bound can be represented for simplicity and unifying purposes as $\left(n_{r}p_{r}-Gp_{r}+1\right)p_{s}$. This upper-bound is obtained from the worst-case formula in sub-case 2.1 when its dominator $(p_{s}$ mod $n_{r}p_{r})=1$.

## Appendix B. Proof of Theorem 6

Recall the proof of Theorem 5 where it was verified that the first successful rendezvous can be achieved within a period whose upper-bound is given by Equation (6). However, to rendezvous over all the *G* common channels, the MTTR upper-bound can be represented after eliminating *G* in (Equation (6)) as (2⌈log${}_{2}L\rceil +3)\times F\left(p_{i},p_{j}\right)$ where $F(p_{i},p_{j})$ is given by:

(A1) $$F\left(p_{i},p_{j}\right)=\left\{\begin{array}{cc} max\{n_{j}p_{j}+2p_{i}-1,\left(n_{i}p_{i}-p_{i}\right)p_{j}\} & \mathrm{if}\;p_{i}<p_{j}\\ max\left\{n_{i},n_{j}\right\}p_{j} & \mathrm{if}\;p_{i}=p_{j}\\ max\{n_{i}p_{i}+2p_{j}-1,\left(n_{j}p_{j}-p_{j}\right)p_{i}\} & \mathrm{if}\;p_{i}>p_{j} \end{array}\right.$$
